# Non-back-drivable clutch based self-locking mechanism of prosthetic joint to improve manipulation stability

**DOI:** 10.3389/fbioe.2024.1385076

**Published:** 2024-05-30

**Authors:** Yang Liu, Yuhui Luo, Ting Xiao, Jiejunyi Liang

**Affiliations:** ^1^ State Key Laboratory of Intelligent Manufacturing Equipment and Technology, Huazhong University of Science and Technology, Wuhan, China; ^2^ School of Art, Soochow University, Suzhou, China; ^3^ Huazhong University of Science and Technology Hospital, Wuhan, China

**Keywords:** prosthesis joint, wrist, non-back-drivable-clutch, backlash, manipulation stability, transient dynamics

## Abstract

During activities of daily living (ADLs), the wrist is mainly engaged in positioning and directing the hand. Researches have demonstrated that restoring wrist mobility can significantly enhance the manipulation ability, reduce body distortion caused by motion compensation, and improve the quality of life for amputees. However, most daily activities, particularly the delicate ones, place high demands on the ability of wrist to maintain a certain rotation angle, also known as non-back-drivable ability, which poses a challenge to the design of prosthetic wrists. To address this issue, various solutions have been proposed, including motor holding brakes, high reduction ratio reducers, and worm gears. However, the motor holding brake only functions after a power outage and cannot continuously prevent torque from the load end. The latter two solutions may alter the transmission ratio, resulting in reduced movement speed and transmission efficiency. Therefore, how to design a miniaturized non-back-drivable mechanism without changing the transmission ratio so that the forearm rotational freedom can be locked at any position for any duration is a problem to be solved in the research of prosthetic wrist designs. This paper presents a line-contact based non-back-drivable clutch (NBDC) that does not cause changes in the transmission ratio, ensuring the motion performance of the prosthetic limb. At the same time, it does not introduce additional friction in the forward transmission process, guaranteeing the overall efficiency. Most importantly, it only allows the torque transmitting from the motor to the load, prevents the load reversely from driving back even in a power failure condition, significantly improving the stability, safety, and comfort. Detailed kinematic and static analyses of the working process has been conducted, and transient dynamics simulation has been performed to verify its effectiveness. Through experiments, it is demonstrated that the self-locking torque of the output end could reach approximately 600 Nmm, and the unlocking torque of the input end is about 80 Nmm, which can be effectively integrated in prosthetic wrist rotation joints, contributing to the performance, safety and energy saving of prosthetic joint systems.

## 1 Introduction

The rotation of the human forearm relies on the ulna and radius turning from a parallel state to an intersecting state. The successful realization of this movement depends on the complete distal radioulnar joint (Neumann, 2016). However, transradial amputation is the most common form of upper limb amputation, which leads to the inevitable destruction of the distal radioulnar joint. As the degree of amputation increases, the rotation ability of the residual limb decreases sharply. Even with a prosthetic hand, the patient’s ADLs are still greatly restricted due to the lack of the most important rotation function of the wrist. In this case, to achieve motion compensation, amputee’s body would inevitably be distorted during manipulation scenarios, which would not only seriously affect the amputee’s comfort, but also bring depression and anxiety to them (Bandara et al., 2014; Semasinghe et al., 2018; Billones et al., 2020). Moreover, it has been discovered that compared with further improving the performance of prosthetic hand, it is more effective to employ a prosthetic wrist to achieve a substantial overall improvement (Montagnani et al., 2015a; Deijs et al., 2016; Bajaj et al., 2019; Lee et al., 2021).

A survey of upper-limb prosthetic users showed that features such as lightweight, durability, and long duration of single charge (more than 12 h) are directly related to whether prosthetic products will be accepted (Mustafa et al., 2006), and among which, lightweight is the most important (Biddiss et al., 2007). Therefore, commercially available prosthetic wrists generally adopt designs those only have a single degree of freedom, retaining the most important rotational freedom to meet the lightweight needs. However, during manipulation tasks, the wrist plays a crucial role in precise positioning the prosthetic hand before the hand comes into contact with the target, and is heavily involved in maintaining stability of the target during the contact, which excessively requires the non-back-drivable ability of the rotating component. To address this issue, various solutions have been proposed, including motor holding brakes, high reduction ratio reducers, and worm gears. However, the motor holding brake only functions after a power outage and cannot continuously prevent torque from the load end (Ann et al., 2000). The latter two solutions may alter the transmission ratio, resulting in reduced movement speed and transmission efficiency. Therefore, how to design a miniaturized non-back-drivable mechanism without changing the transmission ratio so that the forearm rotational freedom can be locked at any position for any duration is a problem to be solved in the research of prosthetic wrists (Controzzi et al., 2010; Kimura et al., 2021; Shi et al., 2021), and is critical to the successful completion of the manipulation task (Gao et al., 2021). In most grasping actions which require to maintain the locking state for a long time, if the actuator lacks a self-locking mechanism, the external load will directly act on the drive motor and drive the prosthetic wrist in the opposite direction, resulting in not only an unstable wrist rotation angle, but also a potential hazard (Guo et al., 2020; Wei et al., 2023). This hazard would be exacerbated in the event of a power failure due to a depleted battery or circuit failure. The unlocked wrist joint will rotate freely under the action of the external load, posing a great danger to amputees. Therefore, prosthetic joints that take into account both miniaturization requirements and self-locking capabilities have become the main development trend, and the key component is the non-back-drivable clutch.

To be specific, the input end of the NBDC is the power system such as the drive motor, and the output end is connected to the hand and the external loads. When the input end receives a motion command and the motor starts to rotate, whether it is clockwise rotation or counterclockwise, as long as the initial torque is greater than the unlocking torque, the transmission system can be activated to achieve efficient power transmission. To the contrary, regardless of whether the external loads apply clockwise or counterclockwise torque to the NBDC, as long as it is within the maximum self-locking torque range, the mechanism will automatically enter the locking state, so that the external loads will not directly affect the drive motor. This feature gives the prosthetic joint the ability to maintain stable grasping at any position for a long time. It also possesses the ability to function without power supply during operation that does not need further rotating, extending the battery life to a certain extent. Therefore, designing advanced NBDC and continuously optimizing its self-locking performance can effectively improve the stability, safety, and endurance of prosthetic joints (Liu et al., 2022).

Researches focusing on how to improve NBDC performance have concluded five optimization directions (Chu et al., 2008; Controzzi et al., 2010; Controzzi et al., 2017; Montagnani et al., 2015b; Hu et al., 2021; Wu et al., 2023): (1) Low cost. In order to meet the large-scale production and implementation of self-locking mechanisms, the cost of related components need to reasonably controlled (Liu et al., 2021) while ensuring the performance and quality (Mota et al., 2023). (2) Simplicity (Zhang et al., 2023), including miniaturization (Ding et al., 2021) and ease of manufacturing and assembly (Controzzi et al., 2010). (3) Safety. Due to the sufficient interaction between humans and prosthetic limbs(Lu et al., 2021), safety is the primary concern in real practice (Hu et al., 2020). If the external load can reversely drive the prosthetic joints, it will cause secondary damage to the amputees. (4) Energy efficiency. How to improve the battery duration of a single charge has always been an important topic in the field of prosthetics (Cirelli et al., 2021; Okafor and Longe, 2022; He et al., 2023). If a single charge cannot satisfy an amputee’s normal use for a day, it will greatly affect its acceptance. As a result, reducing the friction energy loss and inertia would be of great importance (Cirelli et al., 2021). (5) Robustness. As the external load is determined by the actual manipulation tasks, the nominal self-locking torque should be high enough to cover most daily working scenarios.

Focusing on the above five requirements, Marco Controzzi et al. (Controzzi et al., 2010; Controzzi et al., 2017) designed a cylinder-based NBDC and applied it in the SmartHand (Cipriani et al., 2010), enabling it to effectively generate strong gripping force under strict power and weight constraints. Compared with the traditional non-back-drivable mechanism based on worm gears (Kang et al., 2015), this clutch, based on a wedge structure, achieves higher transmission efficiency. Within the load range from 50 Nmm to 150 Nmm, the maximum efficiency of this mechanism is around 0.95. Jun-Uk Chu et al. embedded a self-locking mechanism in the prosthetic finger joint (Chu et al., 2008). The self-locking mechanism adopts a coil spring and cam ball structure to prevent the reverse force of the grasped object on the fingers. However, the springs and cams in this mechanism suffer significant wear during operation, which does not meet the durability requirements of prosthetic devices. Qiqiang Hu et al. (Hu et al., 2021)were inspired by the ratchet spanner and employed an interlock to increase the grip strength of the fingers, enabling them to safely perform high loads and prolonged gripping tasks. However, when the ratchet mechanism is unlocking, it is always in a state of friction with the buckle and is prone to wear. At the same time, there is a certain amount of free travel in the self-locking process, resulting a small deviation at the load end. Xiaofeng Wu et al. (Wu et al., 2023) proposed a compact arc-groove self-locking mechanism with two linear springs embedded in the arc grooves, eliminating the need for additional mounting structures. The whole system is compact, small, and modular, enabling underdriven finger mechanisms to achieve adaptive gripping. However, the groove needs to be regularly filled with an appropriate amount of grease to reduce the impact of friction between the spring and the groove. Federico Montagnani et al. (Montagnani et al., 2015b) designed a non-back-drivable mechanism for miniaturized application scenarios such as finger joints. This mechanism can turn off the power after reaching a stable state, thereby avoiding accidental release of the grasped object, and absorbs the impact generated during grasping. However, this mechanism must also consider the corrosion of lubricants, etc., limiting its application in certain conditions. In summary, for the existing self-locking mechanisms, there are still unsolved problems such as not being able to lock instantly, excessive wear of the system under normal operating conditions, and the need to add grease to the mechanism in a timely manner.

In this study, in order to solve the above problems, a novel NBDC is proposed, which achieves self-locking based on the line-contact between the wedges and the fixed support, and can be applied in prosthetic wrists to realize rotational self-locking. The proposed mechanism consists of a fixed support, a wedge pedestal, four wedges, a pin pedestal and four pins, meriting low cost and simplicity in manufacture and assembly. As to the operating mechanism, it mainly contains three key states: (1) Self-locking state. No matter whether the output end, which is connected to the external loads, moves clockwise or counterclockwise, the loads cannot be transmitted back to the input end. (2) Intermediate state of unlocking. 7° of idle angle exists between unlock state and complete lock state. (3) Unlocking state. When the torque at the input end drives the pin to rotate more than 7° of idle angle and reach 14°, the driving force would be transmitted to the output end, and then the self-locking system start to rotate as a whole. Simulations and Experiments have been conducted and verified that the self-locking structure could resist a reverse torque of 600 Nmm from the output end, and only requires an unlocking torque about 80 Nmm at the input end, making it highly efficient for the implementation in prosthetic wrists or other rotating joints.

The main contributions of this paper are as follows:

(1) A non-back-drivable clutch with self-locking features is designed, which does not change the transmission ratio of the system and can ensure the motion speed of the rotating forearm under the premise of stable manipulation. (2) A wavy spring with suitable stiffness is designed to switch between unlocking and locking modes in the self-locking system, separating the wedges from the support in the unlocking state, reducing the friction and improving the efficiency. (3) Critical working conditions of the self-locking system are analyzed and verified through detailed kinematic analysis, static analysis, transient dynamic simulations and experiments, proving the performance of the proposed system that the self-locking ability reaches 600 Nmm.

The rest of this study is organized as follows. Section 2 introduces the overall design of the new NBDC mechanism, and calculates the theoretical performance of the mechanism through kinematic analysis, static analyses and transient dynamics analysis. Section 3 introduces the experimental platform construction and result analysis of NBDC. Section 4 introduces applications with NBDC embedded in a prosthetic wrist. Section 5 discusses the limitations of this research and possible improvements in the future. The final conclusions can be found in Section 6.

## 2 Materials and methods

**FIGURE 1:**
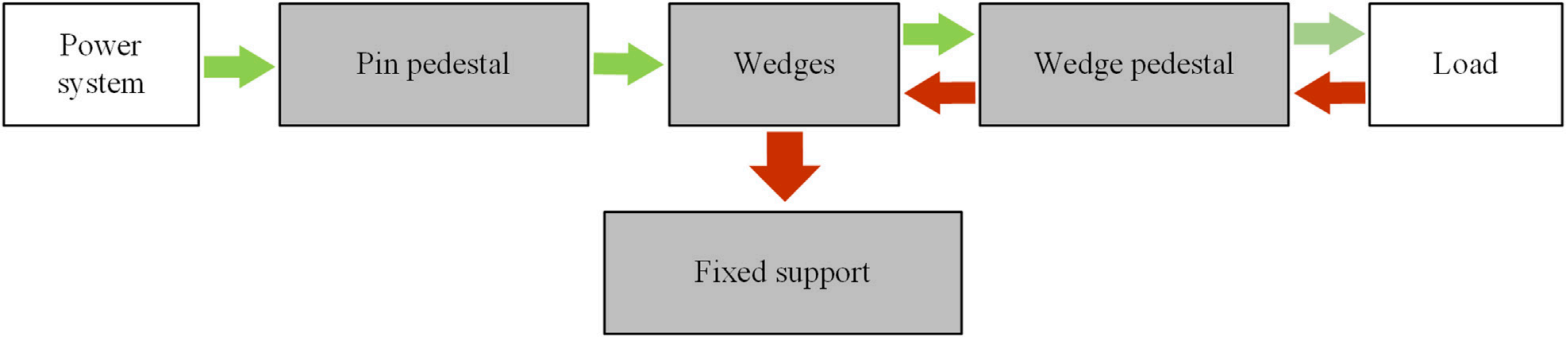
Driving mode (green arrow) and self-locking mode (red arrow) of the NBDC mechanism.

**FIGURE 2:**
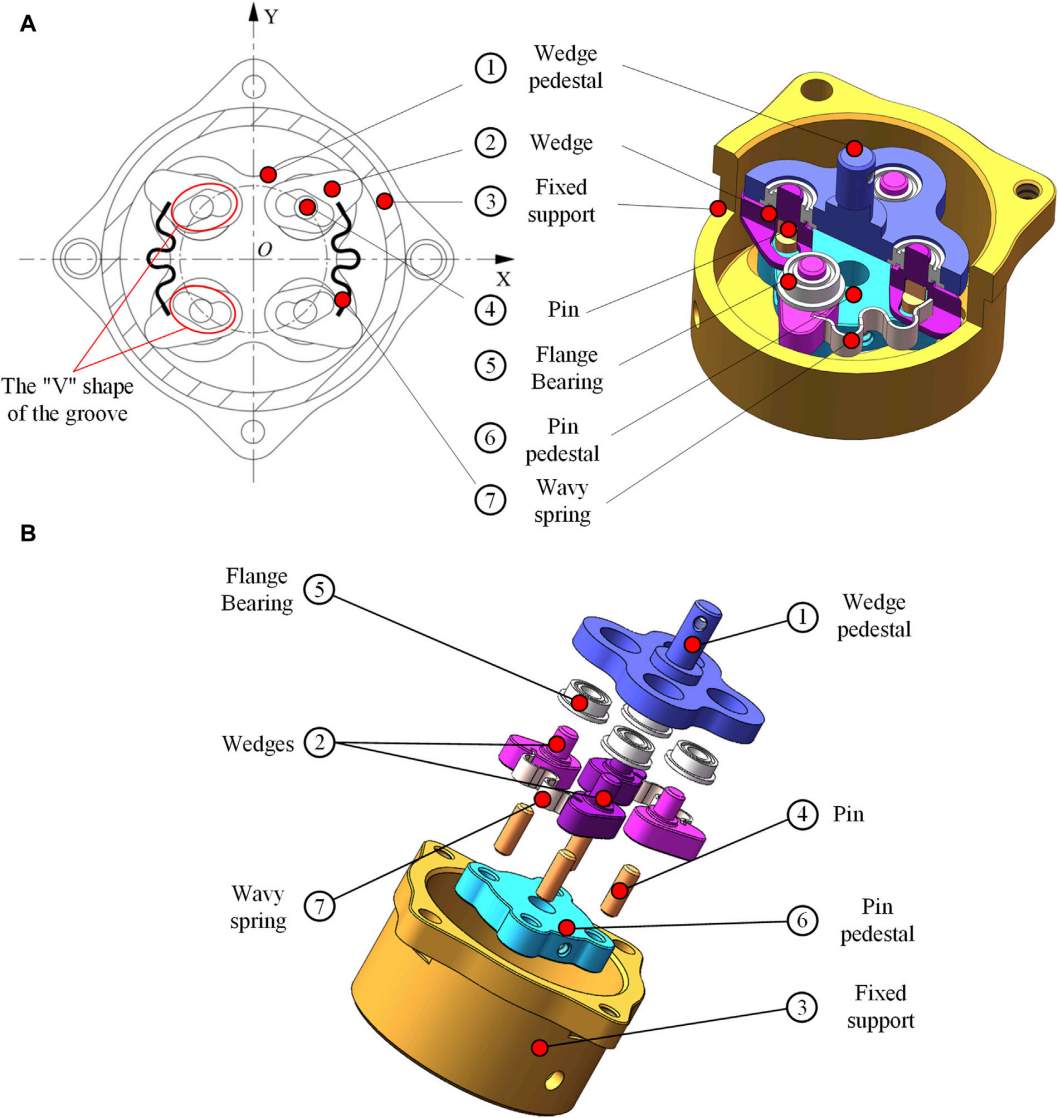
NBDC section view and exploded view. **(A)** Section view. **(B)** Exploded view.

The proposed NBDC based on line-contact self-locking mechanism mainly consists of a fixed support, four wedges, a wedge pedestal, four pins and a pin pedestal. The pin pedestal can be used as the connection to the input end, through which the power system such as the motor could be connected. Four pins are mounted along the circumference of the pin pedestal, the pin drives two sets of wedges to rotate, ultimately generating a self-locking state, an unlocking intermediate state and an unlocking state together with the fixed support (see in Figure 1). Each wedge set is composed of two opposite wedges, connected by a wavy spring in the middle. The shape of each set of wedges is mirror symmetrical. The groove below the wedge connects the pin, and the groove presents a “V” shape. The fixed support is connected to the work platform through bolts, and also connected to the pin pedestal through bearings and pins. Its function is to provide self-locking through the friction between its inner wall and the top arc surface of the wedge in self-locking stage, absorbing the impact from the output end. This clutch is embedded between the wrist rotation motor and the harmonic reducer in practice, with its input end connected to the output shaft of the motor and its output end connected to the harmonic reducer. The specific section view and exploded view of the NBDC mechanism are shown in Figures 2A, B, respectively.

### 2.1 Kinematic analysis

**FIGURE 3:**
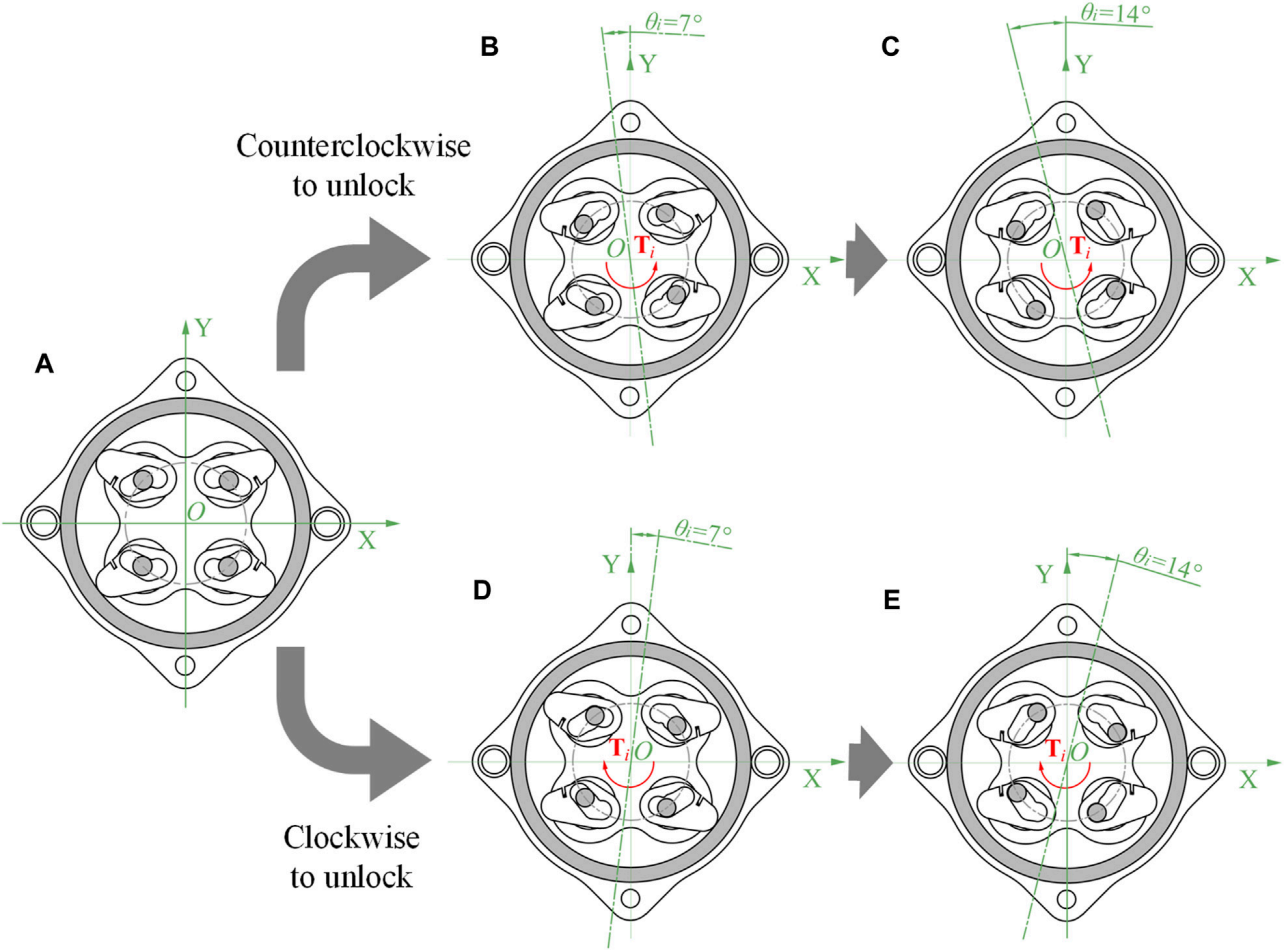
Five key states of the NBDC mechanism (spring not drawn). **(A)** Self-locking state. **(B)** Counterclockwise intermediate state of unlocking. **(C)** Counterclockwise unlocking state. **(D)** Clockwise intermediate state of unlocking. **(E)** Clockwise unlocking state.

In order to clearly describe the operating mechanism of the proposed NBDC, the section views of five key states of the self-locking mechanism are depicted in detail in Figure 3. These section views are drawn along the middle of the axis with the spring neglected, showing the changes of the input end. These five states can be further divided into three categories, which are: (1) Self-locking, as shown in Figure 3A. In this state, the pin pedestal is at zero point, and the pins don’t contact the inner wall of the groove below the wedges. The torque from the output end cannot be transmitted to the input end whether the motion is clockwise nor counterclockwise, and at the same time, the length of the wavy spring is the longest. (2) Intermediate state of unlocking, as shown in Figures 3B, D. The input end drives the pins through the pin pedestal to rotate 7° around the rotation center. Among the four pins, two of them pressure to the inner wall of the groove under the wedge, making it start to rotate. At the same time, the other two pins just reach the contacting point to the surface of the grooves of the above wedge. At this point, in the 2nd and 4th quadrants of Figures 3A, B gap forms between the edge of the wedges and the inner wall of the fixed support. Similarly, in the 1st and 3rd quadrants of Figures 3A, D gap also appears. (3) Unlocking state. The two pins that have been separated from the fixed support move in the groove during the rotation of 7°–14°, and finally reach the bottom of the groove. The other two pins drive their corresponding wedges to start moving, causing them to break away from the fixed support and eventually reach the bottom of the groove, as shown in the 1st and 3rd quadrants in Figure 3C and the 2nd and 4th quadrants in Figure 3E. Thereafter, the torque at the input end can be transmitted directly to the output, and the wavy spring is compressed to its shortest length. When there is no continuous torque input at the input end, the two springs will exert the stored elastic potential energy to press the wedges to the self-locking state again, back to Figure 3A, so that the torque of the external load will be blocked.

**FIGURE 4:**
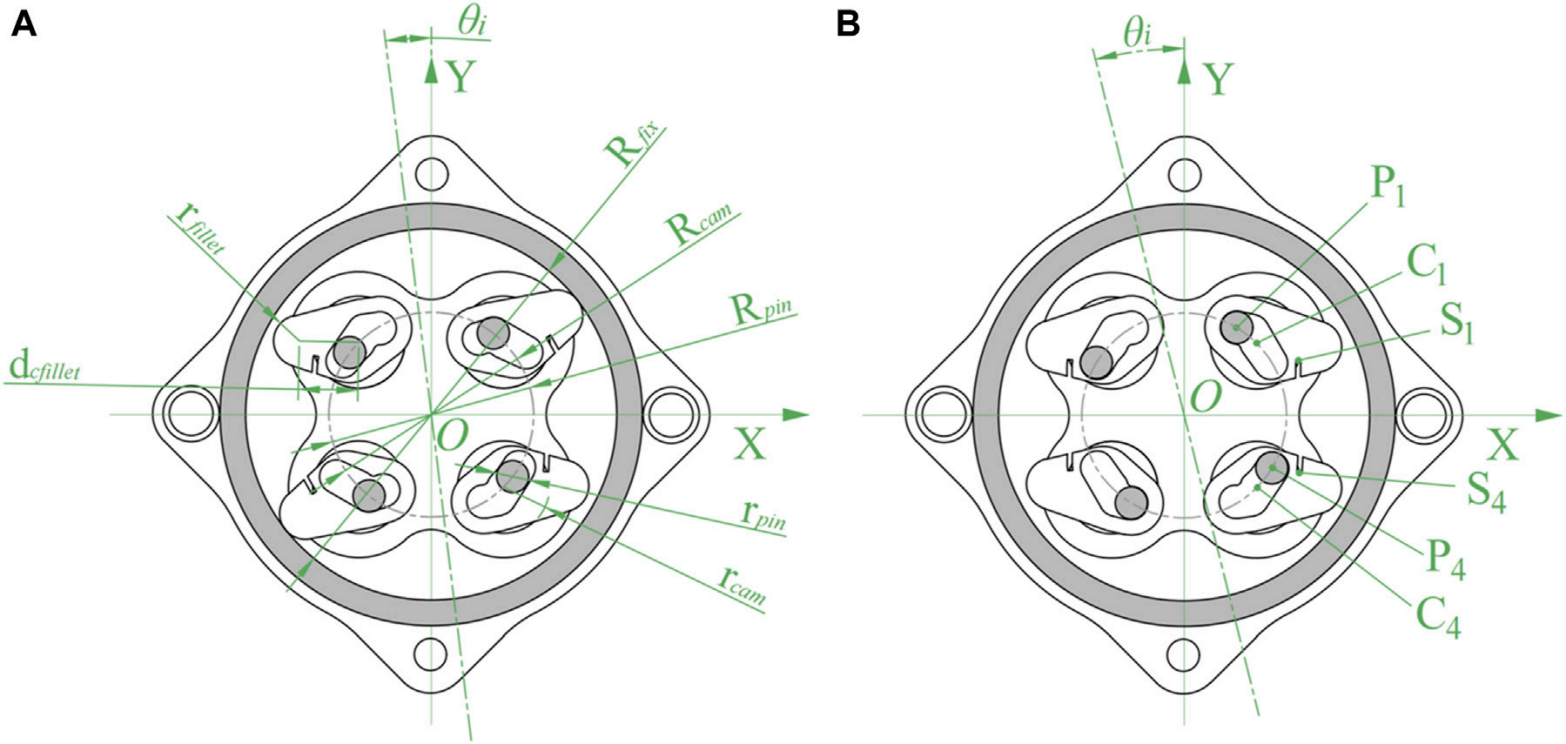
Kinematic analysis of NBDC mechanism (spring not drawn). **(A)** Dimensions. **(B)** Action points.

To investigate the influence of the angle change of the input end on the angle of the wedges in the three working states, kinematic analysis was carried out, taking the 1st and 4th quadrants as examples. As shown in Figure 4A, the inner diameter of the fixed support is $R_{fix}$ , the radius of the pins is $r_{pin}$ , the radius of the circle where the center of the pins is located is $R_{pin}$ , the contact radius between the wedges and the flange bearing is $r_{cam}$ , the radius of the circle where the center of the wedges is located is $R_{cam}$ , and ${\;R}_{pin}$ , $R_{cam}$ have the same value. The angle between the pins is 45°, and the relative angle the pin pedestal rotates to the fixed support is $\theta_{i}$ . In the 1st quadrant, the center of the pins is $P_{1}$ , the center of the wedges is $C_{1}$ , and the action point of the connecting spring is $S_{1}$ . In the 4th quadrant, the center of the pins is $P_{4}$ , the center of the wedges is $C_{4}$ , and the action point of the connecting wavy spring is $S_{4}$ , as shown in Figure 4B.

Taking the counterclockwise movement of the input as an example, the positions of the centers of the pins in the 1st and 4th quadrants at any moment are shown in Eq. 1:

$$\left\{\begin{array}{c} \vec{OP_{1}}=R_{pin}{\left[\cos\left(45{}^{\circ}+\theta_{i}\right)\;\sin\left(45{}^{\circ}+\theta_{i}\right)\right]}^{T}\\ \vec{OP_{4}}=R_{pin}{\left[\cos\left(315{}^{\circ}+\theta_{i}\right)\;\sin\left(315{}^{\circ}+\theta_{i}\right)\right]}^{T} \end{array}\right.$$ (1)

The axis where the wedges are located are uniformly distributed along the center, the angle between them is 90°, and the specific position is related to the rotation angle $\theta_{o}$ of the output end of the wedge pedestal. The position of the center of the wedge in the 1st quadrant and the 4th quadrant are shown in Eq. 2:

$$\left\{\begin{array}{c} \vec{OC_{1}}=R_{cam}{\left[\cos\left(45{}^{\circ}+\theta_{o}\right)\;\sin\left(45{}^{\circ}+\theta_{o}\right)\right]}^{T}\\ \vec{OC_{4}}=R_{cam}{\left[\cos\left(315{}^{\circ}+\theta_{o}\right)\;\sin\left(315{}^{\circ}+\theta_{o}\right)\right]}^{T} \end{array}\right.$$ (2)

When the pin pedestal rotates $\theta_{i}<\theta_{iab}$ , the stage changes from Figures 3A, B, and when the pin pedestal rotates $\theta_{iab}\leq \theta_{i}<\theta_{iac}$ , the stage changes from Figures 3B, C. The most important three topics of these two processes are: (1) The relationship between the rotation angle of the input $\theta_{i}$ , the rotation angles of the wedges in the 1st and 4th quadrants, and the wedge pedestal. (2) The changes in the distances between the 1st (4th) quadrant’s wedge and the inner wall of the fixed support at the rotation angle $\theta_{i}$ ; (3) The effective action length of the wavy spring changes according to the angle $\theta_{i}$ .

(1) According to the geometric relationship that the pin in the 4th quadrant is always tangent to the inner side of the groove of the wedge, it can be seen that the slope between the axis of the wedge and the axis of the pin satisfies the following Eq. 3:

$$k_{\text{CP}}^{4}=\frac{y_{P}^{4}-y_{C}^{4}}{x_{P}^{4}-x_{C}^{4}}=\frac{R_{pin}\sin\left(315{}^{\circ}+\theta_{i}\right)-R_{cam}\sin\left(315{}^{\circ}+\theta_{o}\right)}{R_{pin}\cos\left(315{}^{\circ}+\theta_{i}\right)-R_{cam}\cos\left(315{}^{\circ}+\theta_{o}\right)}$$ (3)

Similarly, the slope between the axis of the wedge and the axis of the pin in the 1st quadrant satisfies Eq. 4:

$$k_{\text{CP}}^{1}=\frac{y_{P}^{1}-y_{C}^{1}}{x_{P}^{1}-x_{C}^{1}}=\frac{R_{pin}\sin\left(45{}^{\circ}+\theta_{i}\right)-R_{cam}\sin\left(45{}^{\circ}+\theta_{o}\right)}{R_{pin}\cos\left(45{}^{\circ}+\theta_{i}\right)-R_{cam}\cos\left(45{}^{\circ}+\theta_{o}\right)}$$ (4)

The initial angle of the wedge in the 1st quadrant is $\theta_{camini}^{1}$ , that in the 4th quadrant is $\theta_{camini}^{4}$ , then relative rotation of the two wedges is Eq. 5:

$$\left\{\begin{array}{c} \theta_{cam}^{1}=\arctan\left(k_{\text{CP}}^{1}\right)-\theta_{camini}^{1}\\ \theta_{cam}^{4}=\arctan\left(k_{\text{CP}}^{4}\right)-\theta_{camini}^{4} \end{array}\right.$$ (5)

**FIGURE 5:**
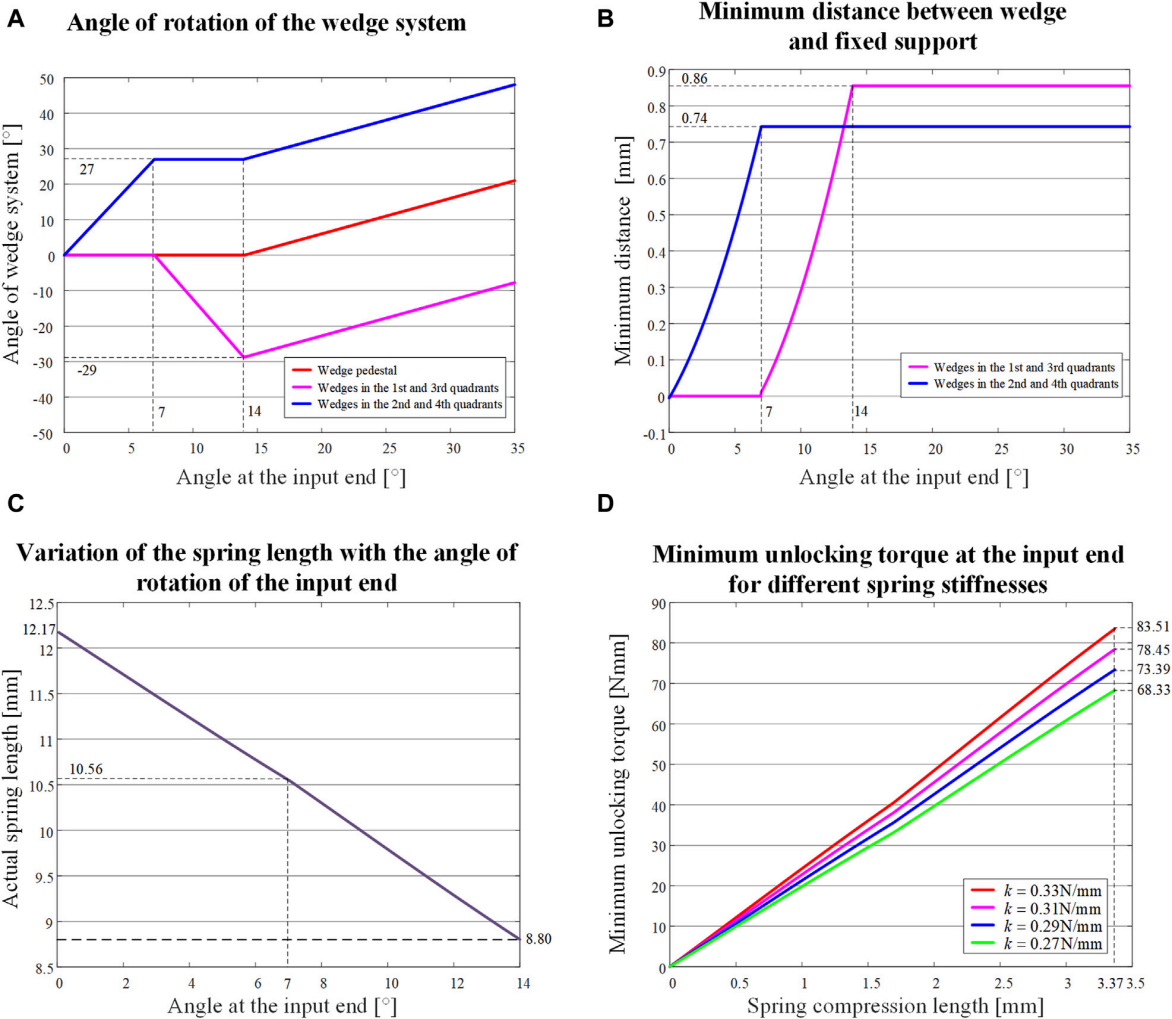
Variation of relevant parameters of the NBDC system with the angle of counterclockwise input. **(A)** Angle of the wedge and the wedge pedestal. **(B)** Minimum distance of the edge of the wedge from the inner wall of the fixed support. **(C)** Length of the spring. **(D)** Minimum unlocking torque of the input end with the compression length of the spring for different spring stiffnesses.

The relationship between the input rotation angle $\theta_{i}$ and the wedge rotation angle $\theta_{cam}^{1}$ , $\theta_{cam}^{4}$ are shown in Figure 5A. When the input angle increases from 0° to 7°, the wedge in the 4th quadrant rotates counterclockwise, the 1st quadrant’s wedge and wedge pedestal stay still. When the input angle increases from 7° to 14°, the wedge in the 4th quadrant continues to rotate counterclockwise, the wedge in the 1st quadrant rotates clockwise, and the wedge pedestal stays still. When the angle of the input continues to increase, the wedge no longer rotates relative to its axis, and the angle of the wedge pedestal as the output end increases by the same amount as that of the input end.

(2) When the input end drives the pin pedestal to rotate, each set of wedges will gradually detach from the inner wall of the fixed support for a certain distance, and the nearest part between the wedges and the fixed support is a section of arc, the radius of which is $r_{fillet}$ , and the distance from the center of the arc to the center of rotation of the wedges is $d_{cfillet\;}$ . In the 1st quadrant, the initial angle of the line between the center of the arc and the center of the wedge is $\theta_{cfillet}^{1}$ . In the 4th quadrant, the initial angle is $\theta_{cfillet}^{4}$ . Therefore, the position of the centers of the arcs at the tips of the two wedges at any instant are in Eq. 6:

$$\left\{\begin{array}{c} \vec{OT_{1}}=\vec{OC_{1}}+d_{cfillet}{\left[\begin{array}{cc} \cos\left(\theta_{cfillet}^{1}+\theta_{cam}^{1}\right) & \sin\left(\theta_{cfillet}^{1}+\theta_{cam}^{1}\right) \end{array}\right]}^{T}\\ \vec{OT_{4}}=\vec{OC_{4}}+d_{cfillet\;}{\left[\begin{array}{cc} \cos\left(\theta_{cfillet}^{4}+\theta_{cam}^{4}\right) & \sin\left(\theta_{cfillet}^{4}+\theta_{cam}^{4}\right) \end{array}\right]}^{T} \end{array}\right.$$ (6)

The distance between the input angle $\theta_{i}$ and the distance between the 1st and 4th quadrants’ wedges and the inner wall of the fixed support $d_{cfix}^{1}$ and $d_{cfix}^{4}$ satisfy Eq. 7:

$$\left\{\begin{array}{c} d_{cfix}^{1}=R_{fix}-\left\|\vec{OT_{1}}\right\|-r_{fillet}\\ d_{cfix}^{4}=R_{fix}-\left\|\vec{OT_{4}}\right\|-r_{fillet} \end{array}\right.$$ (7)

The input angle $\theta_{i}$ against the distance between the wedge and the inner wall of the fixed support is shown in Figure 5B. The maximum value of the minimum distance between the wedge and the inner wall of the fixed support in the 1st and 3rd quadrants is 0.86 mm, and the maximum value of the minimum distance between the wedge and the inner wall of the fixed support in the 2nd and 4th quadrants is 0.74 mm, which ensures that the relative motion in the unlocked state generates as little frictional wear as possible.

(3) During this process, the spring would be compressed to its shortest length. Taking the wavy spring between the wedges in the 1st and 4th quadrants as an example, the positions of the connection point between the spring and the two wedges at any time are shown in Eq. 8:

$$\left\{\begin{array}{c} \vec{OS_{1}}=\vec{OC_{1}}+d_{\text{CS}}{\left[\begin{array}{cc} \cos\left(\theta_{\text{CS}}^{1}+\theta_{cam}^{1}\right) & \sin\left(\theta_{\text{CS}}^{1}+\theta_{cam}^{1}\right) \end{array}\right]}^{T}\\ \vec{OS_{4}}=\vec{OC_{4}}+d_{\text{CS}}{\left[\begin{array}{cc} \cos\left(\theta_{\text{CS}}^{4}+\theta_{cam}^{4}\right) & \sin\left(\theta_{\text{CS}}^{4}+\theta_{cam}^{4}\right) \end{array}\right]}^{T} \end{array}\right.$$ (8)

where the distance from the connection point of the top of the wavy spring to the center of rotation of the wedge is $d_{\text{CS}}$ , and the initial angles of the line connecting the connection point to the centers of rotation of the wedge in the 1st and 4th quadrants are $\theta_{\text{CS}}^{1}$ and $\theta_{\text{CS}}^{4}$ . The length of the wavy spring can be calculated from the distance between the two connection points shown in Eq. 9:

$$L_{s}=\left\|\vec{OS_{1}}-\vec{OS_{4}}\right\|=\left\|\vec{S_{4}S_{1}}\right\|$$ (9)

The relationship of the input angel $\theta_{i}$ against the length of the spring $L_{s}$ (the original length of the spring is $L_{s0}$ ) is shown in Figure 5C. As the input angle increases, the length of the wavy spring is shortened from 12.17 mm to 10.56 mm in the first stage of 0°–7°, and further shortened from 10.56 mm to 8.80 mm in the second stage of 7°–14°, where the total shortage was 3.37 mm.

In the phase after Figure 3C, the pins no longer move with respect to the wedges, and the wedges no longer continue to rotate with respect to its own axis. The wedge pedestal at the output end follows the pin pedestal at the input end by the same angle $\theta_{o}=\theta_{i}-\theta_{iac}$ .

### 2.2 Static analysis

The main function of the NBDC is to always remain locked when the load on the output end changes and to be able to unlock smoothly when power is supplied from the input end. In order to ensure the proper functioning of the mechanism, it is necessary to carry out force analysis for two different force conditions, which are torque $T_{i}$ applied to the input end, and load torque $T_{o}$ applied to the output end. When the self-locking requirements are met, the minimum friction coefficient between the wedge and the fixed support would be investigated, when the unlocking requirements are met, the reasonable stiffness of the wavy spring would be revealed.

#### 2.2.1 Unlocking status analysis

Taking the counterclockwise input as an example, the initial state is the self-locking state shown in Figure 3A. When the system is subjected to counterclockwise input torque $T_{i}$ , the pin pedestal drives the pins to rotate counterclockwise, i.e., $C_{i}$ , i = 1, 2, 3, 4 rotates counterclockwise around the point O . In the 1st quadrant, the pin is not in contact with the groove of the wedge and can rotate freely. In the 4th quadrant, the pin is in contact with the groove of the wedge, generating a pressure $F_{pin}^{4}$ , which is given by the input torque $T_{i}$ in Eq. 10:

$$T_{i}=2\left(\vec{OC_{4}}\times F_{pin}^{4}\right)$$ (10)

The center of the pin $P_{4}$ is rotating along its trajectory, and the friction force $F_{T}^{4}$ ( $F_{T}^{4}=\mu_{cp}\cdot F_{pin}^{4}$ ) is acting at the tangent point of the wedge $Q_{4}$ (moving along the inner wall of the groove of the wedge), $\mu_{cp}$ is the coefficient of friction between the surface of the pin and the inner surface of the groove of the wedge (when the pin is made of stainless steel, the wedge is made of 7,075 aluminum alloy, $\mu_{cp}$ = 0.4–0.7, take $\mu_{cp}$ = 0.6). $F_{T}^{4}$ exerts a torque $\vec{C_{4}Q_{4}}\times F_{T}^{4}$ on the center of the wedge $C_{4}$ . The wedge tends to rotate counterclockwise around the point $C_{4}$ . We need to investigate whether friction exists at the point $A_{4}$ , which is contacted between the wedge and the inner wall of the fixed support. The point $T_{4}$ is the center of the top arc of the wedge. Connect $\vec{C_{4}T_{4}}$ and extend it, intersecting the top arc of the wedge at point $B_{4}$ . Since both $\vec{T_{4}B_{4}}$ and $\vec{T_{4}A_{4}}$ are arc radius, $\left\|\vec{T_{4}B_{4}}\right\|=\left\|\vec{T_{4}A_{4}}\right\|$ . At the same time points $C_{4}$ , $T_{4}$ , $A_{4}$ are not collinear, therefore, Eq. 11 holds:

$$\left\|\vec{C_{4}B_{4}}\right\|=\left\|\vec{C_{4}T_{4}}+\vec{T_{4}B_{4}}\right\|=\left\|\vec{C_{4}T_{4}}\right\|+\left\|\vec{T_{4}B_{4}}\right\|=\left\|\vec{C_{4}T_{4}}\right\|+\left\|\vec{T_{4}A_{4}}\right\|>\left\|\vec{C_{4}T_{4}}+\vec{T_{4}A_{4}}\right\|=\left\|\vec{C_{4}A_{4}}\right\|$$ (11)

i.e., $\left\|\vec{C_{4}B_{4}}\right\|>\left\|\vec{C_{4}A_{4}}\right\|$ (identically $\left\|\vec{C_{1}B_{1}}\right\|>\left\|\vec{C_{1}A_{1}}\right\|$ in the 1st quadrant), and the distance from any point on the arc below point $C_{4}$ to point $A_{4}$ is less than $\left\|\vec{C_{4}A_{4}}\right\|$ . Therefore, when the wedge is rotated counterclockwise, the top arc of the wedge is disengaged from the inner wall of the fixed support and there is no friction.

The spring between the two wedges $\left\|\vec{S_{1}S_{4}}\right\|$ is compressed and shortened due to the counterclockwise rotation of the wedges. In the 1st quadrant, the wedge tends to move counterclockwise due to the action of $F_{s}^{1}$ , but from the above derivation, $\left\|\vec{C_{1}B_{1}}\right\|>\left\|\vec{C_{1}A_{1}}\right\|$ , so the wedge cannot rotate counterclockwise. As the spring deformation $\Delta s=L_{s}^{\theta_{iab}}-L_{s}^{0^{\circ }}$ becomes larger, $F_{s}^{4}$ produces a clockwise torque $\vec{C_{4}S_{4}}\times F_{s}^{4}$ in the 4th quadrant, where $\left\|F_{s}^{4}\right\|=k\cdot \left|\Delta s\right|=k\cdot \left|\left(L_{s}^{\theta_{iab}}-L_{s}^{0^{\circ }}\right)\right|$ , k is the stiffness of the wavy spring. When the pin pedestal is rotated counterclockwise by 7°, as shown in Figure 3B, the pins in the 1st and 3rd quadrants start to contact the inner groove surface of their wedges, and the deformation of the springs reaches the maximum at this stage, at which time the total torque on the wedges in the 4th quadrant is ${\;M}_{cam}^{i}$ , shown in Eq. 12:

$$M_{cam}^{i}=\vec{C_{4}Q_{4}}\times F_{T}^{4}+\vec{C_{4}S_{4}}\times F_{s}^{4}$$ (12)

Since the unlocking of the mechanism needs to keep the 4th quadrant wedge moving counterclockwise around the point $C_{4}$ , specify the counterclockwise torque as positive and the clockwise torque as negative, it needs to satisfy Eq. 13:

$$\left\|M_{cam}^{4}\right\|=\left\|\vec{C_{4}Q_{4}}\times F_{T}^{4}\right\|-\left\|\vec{C_{4}S_{4}}\times F_{s}^{4}\right\|>0$$ (13)

Substituting Eq. 10 into Eq. 13 yields Eq. 14:

$$\left\|F_{pin}^{4}\right\|>\frac{k\cdot \left\|\vec{C_{4}S_{4}}\right\|}{\mu_{cp}\cdot r_{pin}}\cdot \left(L_{s}^{0^{\circ }}-L_{s}^{7^{\circ }}\right)$$ (14)

Define the angle between $\vec{OC_{i}}$ and $F_{pin}^{i}$ , i = 1, 2, 3, 4 as $\gamma_{\text{CP}}^{i}$ , $\vec{OC_{i}}$ and $F_{s}^{i}$ , i = 1, 2, 3, 4 as $\gamma_{\text{CS}}^{i}$ . In the process from Figures 3A, B, the variation of $\gamma_{\text{CP}}^{4}$ ranges from 17° to 0°, then $T_{i}$ can be represented in Eq. 15:

$$\left\|T_{i}\right\|=2\times \left(\left\|\vec{OC_{4}}\right\|\cdot \left\|F_{pin}^{4}\right\|\cdot \sin\left(\gamma_{\text{CP}}^{4}\right)\right)$$ (15)

Substituting Eq. 15 into Eq. 14 yields Eq. 16:

$$\left\|T_{i}\right\|>\frac{2k}{\mu_{cp}}\cdot \frac{R_{cam}\cdot d_{\text{CS}}}{r_{pin}}\cdot \left(L_{s}^{0^{\circ }}-L_{s}^{7^{\circ }}\right)\cdot \sin\left(\gamma_{\text{CP}}^{4}\right)\cdot \sin\left(\gamma_{\text{CS}}^{4}\right)$$ (16)

Due to $\sin\left(\gamma_{\text{CP}}^{4}\right)\cdot \sin\left(\gamma_{\text{CS}}^{4}\right)\leq 1$ , Eq. 17 can be got:

$$\left\|T_{iab}^{cri}\right\|=\frac{2k}{\mu_{cp}}\cdot \frac{R_{cam}\cdot d_{\text{CS}}}{r_{pin}}\cdot \left(L_{s}^{0^{\circ }}-L_{s}^{7^{\circ }}\right)$$ (17)

**FIGURE 6:**
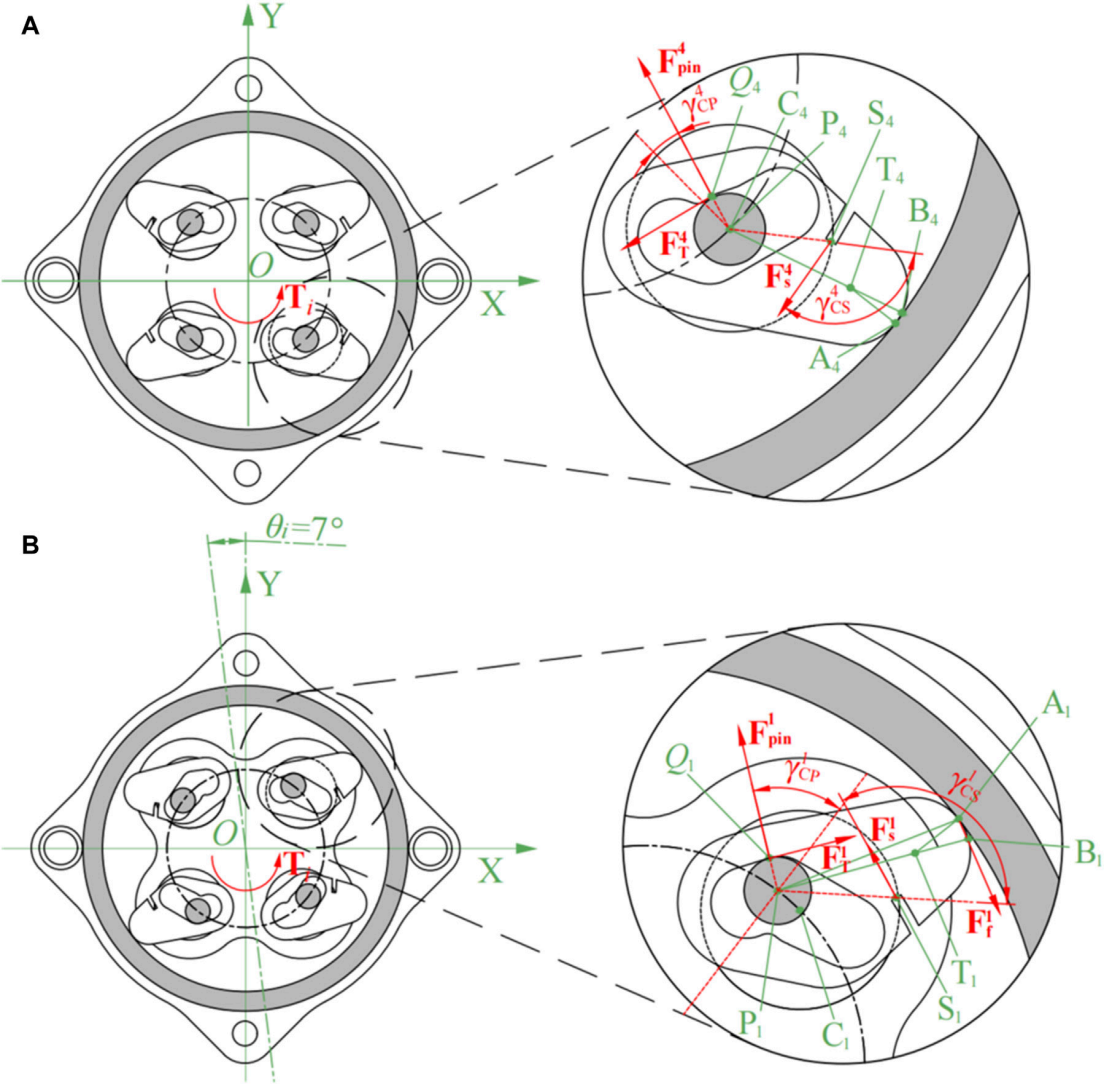
Mechanical analysis of the unlocking process (spring not drawn). **(A)** The initial counterclockwise rotate. **(B)** The input rotated 7° counterclockwise.

The minimum unlocking torque at the input for the process from Figures 3A, B can be calculated as $T_{iab}^{cri}$ , and the magnitude of this value depends on the stiffness k of the wavy spring, provided that the dimensions of the structure and the material of the part are determined. The static analysis of this process is shown in Figure 6A.

When the input is in the intermediate state of counterclockwise unlocking, the pins in the 1st and 3rd quadrants are just touching the inner wall of the wedge grooves. At this point, the pins exert positive pressure on the inner wall only in the 1st and 3rd quadrants $F_{pin}^{1}$ and $F_{pin}^{3}$ (the $F_{pin}^{2}$ and $F_{pin}^{4}$ disappear in the 2nd and 4th quadrants because the pins’ trajectory coincides with the groove trajectory). There is a tendency for the pins in the 1st and 3rd quadrants to slide counterclockwise, generating a corresponding friction force at the point of contact, and the wedges are subjected to a corresponding reverse friction force $F_{T}^{1}$ and $F_{T}^{3}$ ( $F_{T}^{1}=\mu_{cp}\cdot F_{pin}^{1}$ ; $F_{T}^{3}=\mu_{cp}\cdot F_{pin}^{3}$ ). Due to the symmetry of the system, the input torque is $T_{i}=2\left(\vec{OC_{i}}\times F_{pin}^{1}\right)$ . Since $\left\|\vec{C_{1}B_{1}}\right\|>\left\|\vec{C_{1}A_{1}}\right\|$ , when the wedge tends to rotate clockwise around the point $C_{1}$ , point $A_{1}$ detaches from the wall of the fixed support. At the same time, the distance from any point above it to the point $C_{1}$ is smaller than $\left\|\vec{C_{1}A_{1}}\right\|$ , the friction $F_{f}^{1}$ disappears. Smooth unlocking only requires the fulfilment of $\left\|M_{cam}^{1}\right\|=\left\|\vec{C_{1}Q_{1}}\times F_{T}^{1}\right\|-\left\|\vec{C_{1}S_{1}}\times F_{s}^{1}\right\|>0$ , as shown in Eq. 18:

$$\left\|F_{pin}^{1}\right\|>\frac{k\cdot d_{\text{CS}}}{\mu_{cp}\cdot r_{pin}}\cdot \left(L_{s}^{0^{\circ }}-L_{s}^{7^{\circ }}\right)\cdot \sin\left(\gamma_{\text{CS}}^{1}\right)$$ (18)

Owing to Eq. 19:

$$\left\|T_{i}\right\|=\left\|2\left(\vec{OC_{i}}\times F_{pin}^{1}\right)\right\|=2\times \left(R_{cam}\cdot \left\|F_{pin}^{1}\right\|\cdot \sin\left(\gamma_{\text{CP}}^{1}\right)\right)$$ (19)

Substituting Eq. 19 into Eq. 18 yields Eq. 20:

$$\left\|T_{i}\right\|>\frac{2k}{\mu_{cp}}\cdot \frac{R_{cam}\cdot d_{\text{CS}}\cdot \left(L_{s}^{0^{\circ }}-L_{s}^{7^{\circ }}\right)}{r_{pin}}\cdot \sin\left(\gamma_{\text{CP}}^{1}\right)\cdot \sin\left(\gamma_{\text{CS}}^{1}\right)$$ (20)

Due to $\sin\left(\gamma_{\text{CP}}^{1}\right)\cdot \sin\left(\gamma_{\text{CS}}^{1}\right)\leq 1$ and $L_{s}^{14^{\circ }}<L_{s}^{7^{\circ }}$ , we have Eq. 21:

$$\left\|T_{iac}^{cri}\right\|=\frac{2k}{\mu_{cp}}\cdot \frac{R_{cam}\cdot d_{\text{CS}}\cdot \left(L_{s}^{0^{\circ }}-L_{s}^{14^{\circ }}\right)}{r_{pin}}$$ (21)

For smooth unlocking, the input torque should be at least $T_{iac}^{cri}$ and $\left\|T_{iac}^{cri}\right\|>\left\|T_{iab}^{cri}\right\|$ . Due to the symmetry of the mechanism, $\left\|T_{iac}^{cri}\right\|=\left\|T_{iae}^{cri}\right\|$ , $T_{iae}^{cri}$ is the minimum torque for clockwise unlocking of the input. Substituting the relevant parameters, the relationship between the torque and the stiffness k (Nmm) of the wavy spring can be found as in Eq. 22:

$$\left\|T_{iac}^{cri}\right\|=\left\|T_{iae}^{cri}\right\|=k\cdot 253.06\;\left(\text{Nmm}\right)$$ (22)

**FIGURE 7:**
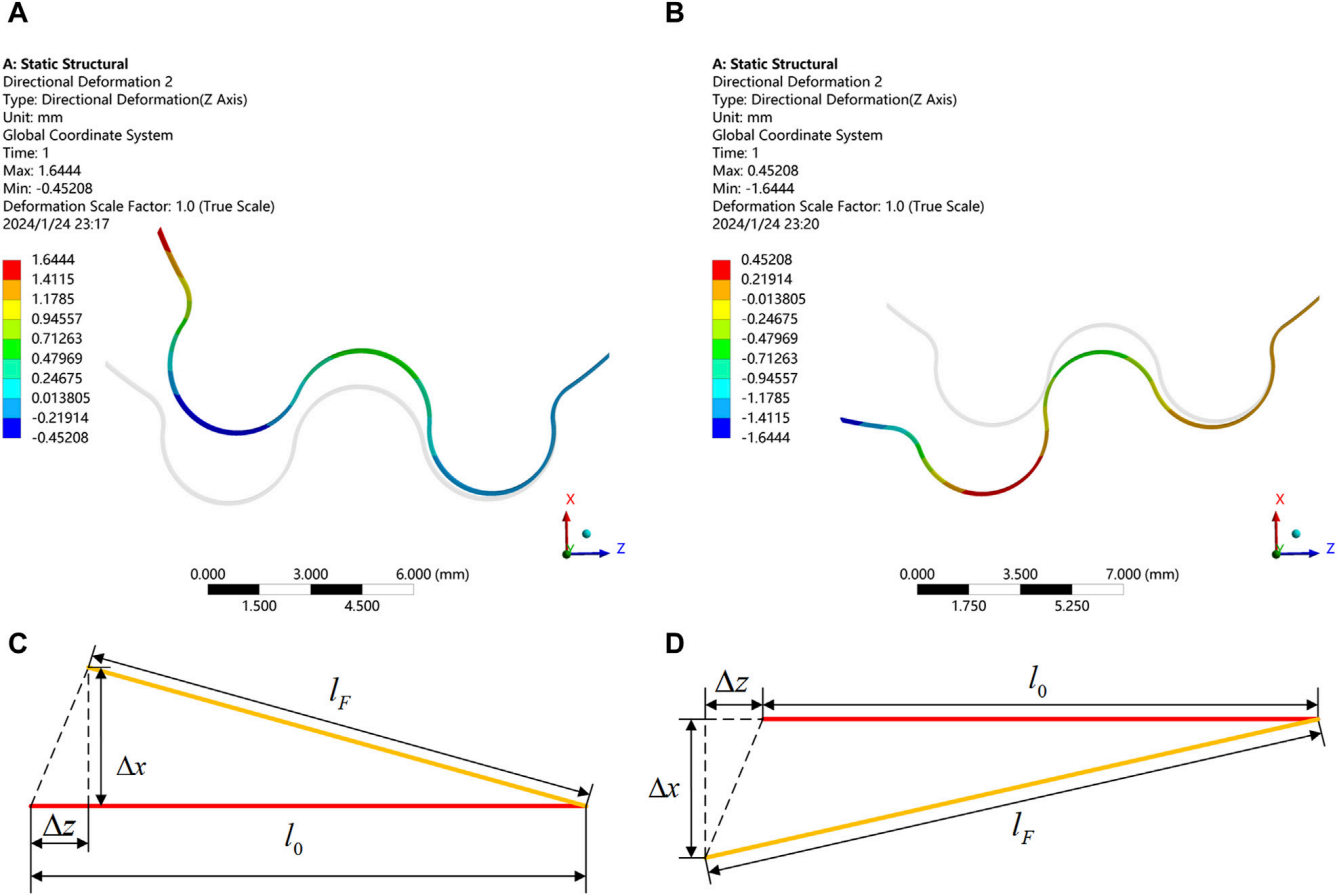
Static simulation of wavy spring in compression and tension. **(A)** Compression displacement of spring in Z-direction. **(B)** Tension displacement of spring in Z-direction. **(C)** Compression variation of spring in Z-direction. **(D)** Tension variation of spring in Z-direction.

After the aforementioned process, the pin pedestal continues to rotate counterclockwise by 7° as shown from Figures 3B, C, and the static analysis is shown in Figure 6B. In order to investigate the influence of the thickness and material on the stiffness of wavy spring, simulations were carried out using hydrostatic simulation software. The specific analytical settings for the wavy spring’s thickness was 0.1 mm, and the material was 301 stainless steels. The horizontal direction was set to be the Z-axis. In Figure 7A, the right end of the wavy spring was fixed and a compression force of 0.5 N was applied to the left end of the wavy spring. In Figures 7A, B pulling force of 0.5 N was applied to the left end of the wavy spring with the right end fixed. It can be seen from Figures 7A, B that the wavy spring was both deformed by 1.6444 mm under the load of 0.5 N in either direction. To better convey the idea, the simplified the model in these two cases are shown in Figures 7C, D.

The stiffness of the spring is calculated with Eq. 23:

$$k=\frac{F}{\Delta z}=\frac{0.5}{1.6444}=0.304\;N/\text{mm}$$ (23)

Substituting Eq. 22 into Eq. 23 yields Eq. 24:

$$\left\|T_{iac}^{cri}\right\|=\left\|T_{iae}^{cri}\right\|\approx 0.304\times 253.06=76.95\text{ Nmm}$$ (24)

When considering the spring manufacturing accuracy, the stiffness variation range is taken as 0.270–0.330 N/mm, the variation of the minimum unlocking torque of the system with the compression length of the spring is shown in Figure 5D. For smooth unlocking, the minimum unlocking torque should be around 65–85 Nmm.

#### 2.2.2 Self-locking status analysis

**FIGURE 8:**
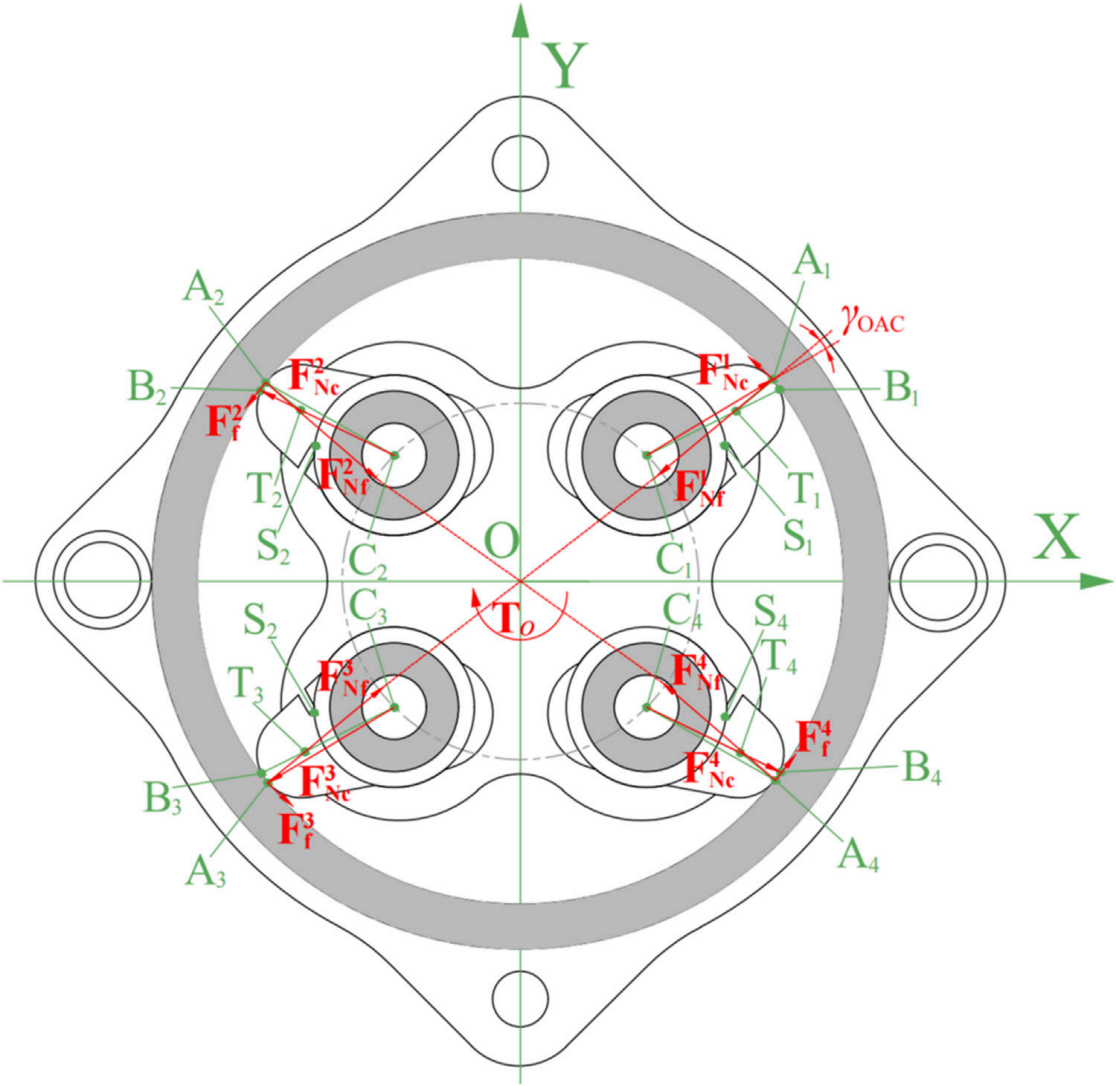
Static analysis of the system with counterclockwise loading torque at the output end under self-locking stage (spring not drawn).

When the input torque is less than the critical starting torque $T_{iac}^{cri}$ or $T_{iae}^{cri}$ , the spring releases to drive the two wedges to contact the inner wall of the fixed support, at this point, the system enters the self-locking state. In this state, the output end applies force to the wedge through the wedge pedestal, and the mechanical analysis of the output end is shown in Figure 8.

Since the wedge pedestal provide the rotational torque through the flange bearings, the forces acting on each of the four wedges are $F_{Nc}^{i}$ , i = 1, 2, 3, 4, $T_{o}=2\left(\vec{OC_{1}}\times F_{Nc}^{1}+\vec{OC_{4}}\times F_{Nc}^{4}\right)$ . At the top of the wedge are reaction force $F_{Nf}^{i}$ , i = 1, 2, 3, 4 from the wall of the fixed support and the corresponding frictions are $F_{f}^{i}$ , i = 1, 2, 3, 4. In the 2nd and 4th quadrants, the combined force of $F_{Nc}^{i}$ , $F_{Nf}^{i}$ , $F_{f}^{i}$ , i = 2, 4, acts on points $C_{2}$ and $C_{4}$ , so that the wedges tend to rotate counterclockwise with respect to their respective centers of rotation. Taking the 4th quadrant wedge as an example, when the wedge is rotated by a certain angle, the wedge is no longer tangent to the fixed support wall, and $F_{Nf}^{4}$ and $F_{f}^{4}$ disappear, the wedge would return into a steady state. Assume the system reaches a self-locking state, the angle between $\vec{OA_{1}}$ and $\vec{C_{1}A_{1}}$ is $\gamma_{\text{OAC}}$ , we get Eq. 25:

$$\left\{\begin{array}{l} \left\|F_{Nf}^{1}\right\|=\left\|F_{Nc}^{1}\right\|\cdot \cos\left(\gamma_{\text{OAC}}\right)\\ \left\|F_{f}^{1}\right\|=\left\|F_{Nc}^{1}\right\|\cdot \sin\left(\gamma_{\text{OAC}}\right) \end{array}\right.$$ (25)

At this point, friction $\left\|F_{f}^{1}\right\|$ is less than maximum friction ${\left\|F_{f}^{1}\right\|}_{\max}=\mu_{cf}\cdot \left\|F_{Nf}^{1}\right\|$ , i.e., $\left\|F_{f}^{1}\right\|<\mu_{cf}\cdot \left\|F_{Nf}^{1}\right\|$ . Substitute into Eq. 25, and we can get Eq. 26:

$$\mu_{cf}>\tan\left(\gamma_{\text{OAC}}\right)\approx 0.158$$ (26)

It can be seen that when the coefficient of friction between the wedge and the inner wall of the fixed support meets the requirements, regardless of the torque applied in any direction at the output end, it will cause instant self-locking. However, when the load torque is too large, causing excessive deformation of the curved surface at the top of the wedge, i.e., $\left\|C_{i}B_{i}\right\|\leq \left\|C_{i}A_{i}\right\|$ , the self-locking will fail, and this process would be demonstrated in the following simulations and experiments.

### 2.3 Transient dynamic simulations and analysis

In order to verify the accuracy of the above theoretical analysis, simulation work was also carried out. The platform was Workbench of ANSYS 2020R2, with the transient dynamics analysis module. The transient dynamics model can effectively analyze the impact of external shocks on the system. On the one hand, it can be verified whether the system can be unlocked when the input end is rotated in the self-locking state. On the other hand, the system self-locking performance of the output end under load can be verified. To reduce the complexity of the iteration without affecting the simulation results, the spring force was simplified to be the maximum force at the compression extreme. This is because the minimum unlocking moment of the system depends on the stiffness k of the wavy spring.

**FIGURE 9:**
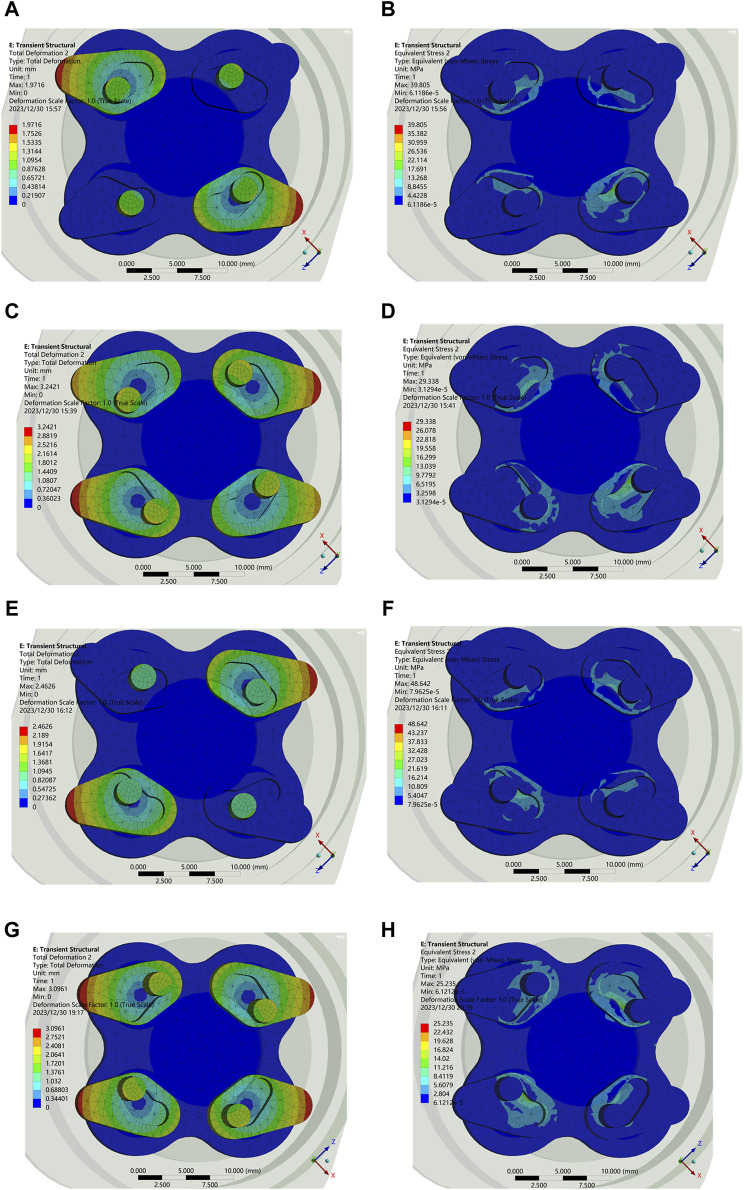
Displacement and stress. **(A)** Displacement at 7° counterclockwise. **(B)** Stress at 7° counterclockwise. **(C)** Displacement at 14° counterclockwise. **(D)** Stress at 14° counterclockwise. **(E)** Displacement at 7° clockwise. **(F)** Stress at 7° clockwise. **(G)** Displacement at 14° clockwise. **(H)** Stress at 14° clockwise.

In order to verify the unlocking performance of the input end, the displacement and stress of the wedge when rotating by 7° and 14° in counterclockwise and clockwise were investigated, respectively. Figures 9A, E describe the counterclockwise and clockwise unlocking intermediate states (rotate by 7°) respectively, and the corresponding stress values are shown in Figures 9B, F. The stress maxima are basically the same, which are all lower than 50 MPa, within the nominal value of 7,075 aluminum alloy. The simulation results are consistent with the design in Figures 3B, D. Similarly, Figures 9C, G are the displacement for 14° counterclockwise and clockwise rotations of the input, and their corresponding stress diagrams are in Figures 9D, H, which is also in accordance with Figures 3C, E.

**FIGURE 10:**
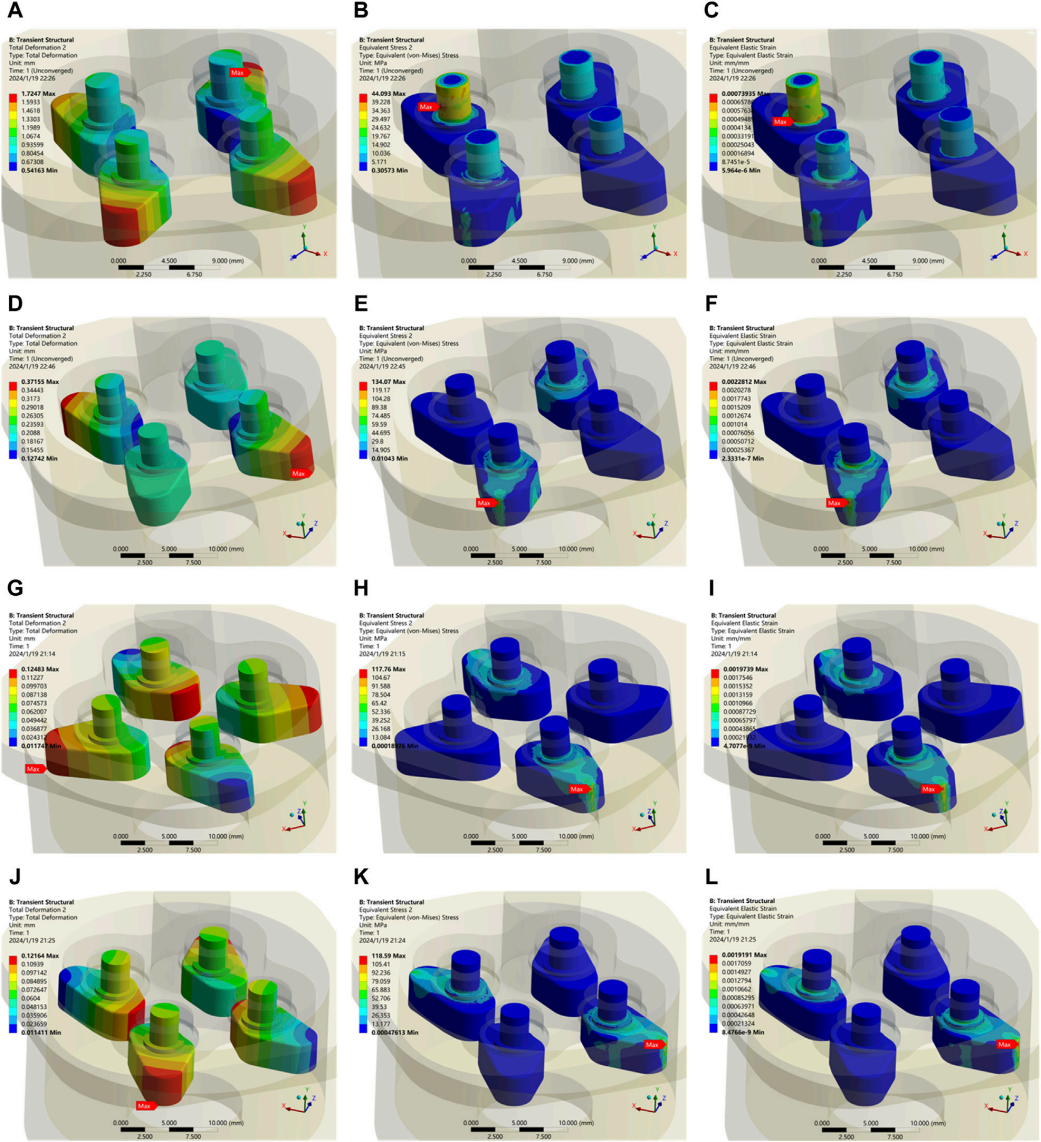
Transient dynamic analysis of the output end with applied load. **(A)** Self-locking failure with displacement of the wedge. **(B)** Stress in the wedge in self-locking failure. **(C)** Strain in the wedge in self-locking failure. **(D)** Displacement of the wedge in the state under $\mu_{cf}$ = 0.16, $T_{o}$ = 600 Nmm. **(E)** Stress in the wedge under $\mu_{cf}$ = 0.16, $T_{o}$ = 600 Nmm. **(F)** Displacement of the wedge under $\mu_{cf}$ = 0.16, $T_{o}$ = 600 Nmm. **(G)** Displacement of the wedge under $\mu_{cf}$ = 0.25, $T_{o}$ = 600 Nmm. **(H)** Stress in the wedge under $\mu_{cf}$ = 0.25, $T_{o}$ = 600 Nmm. **(I)** Displacement of the wedge under $\mu_{cf}$ = 0.25, ${\;T}_{o}$ = 600 Nmm. **(J)** Displacement of the wedge under $\mu_{cf}$ = 0.25, $T_{o}$ = −600 Nmm. **(K)** Stress in the wedge under $\mu_{cf}$ = 0.25, $T_{o}$ = −600 Nmm. **(L)** Displacement of the wedge under $\mu_{cf}$ = 0.25, $T_{o}$ = −600 Nmm.

In order to verify the self-locking performance of the output end, the arc surface strain at the top of the wedge is set to be ε = 0.2%, which is used as the boundary of self-locking failure (Karbalaei Akbari et al., 2013). The performance of clockwise and counterclockwise loads on the shaft of the wedge pedestal is verified respectively. The load was set from 100 Nmm to 800 Nmm with a step of 100 Nmm. When $\mu_{cf}$ = 0.15 and $T_{o}$ = 100 Nmm, the self-locking experience a failure, and all four wedges are displaced, as shown in Figure 10A. The stress is concentrated at the cylindrical surface where the wedge is in contact with the flange bearing, and the maximum strain is also at 0.074%, as shown in Figures 10B, C, respectively. When $\mu_{cf}$ = 0.16 and $T_{o}$ = 600 Nmm, the self-locking function well, justifying Eq. 26. However, at this point, the wedges in the 2nd and 4th quadrants complete the self-locking process, the maximum stress value is 134.07 MPa, the strain is 0.228% > 0.2%, as shown in Figures 10D–F, respectively. It can be concluded that $\mu_{cf}$ = 0.16 is not the ideal friction coefficient for the self-locking system.

For further investigation, the range of $\mu_{cf}$ is set to be from 0.20 to 0.35 with a step size of 0.05. When $T_{o}$ = 600 Nmm (positive is counterclockwise, negative is clockwise) and $\mu_{cf}$ = 0.25, the corresponding displacements, stresses and strains are shown in Figures 10G–I, the displacement of the top of the wedges in the 2nd and 4th quadrants is 0.0117 mm, the system completes self-locking with a strain of 0.197% and a maximum stress of 117.76 MPa. When $T_{o}$ = −600 Nmm and $\mu_{cf}$ = 0.25, the corresponding displacements, stresses and strains are shown in Figures 10J, K, L, the displacement of the top of the wedges in the 1st and 3rd quadrants is 0.0114 mm, the system completes self-locking with a strain of 0.192% and a maximum stress 118.59 MPa.

**FIGURE 11:**
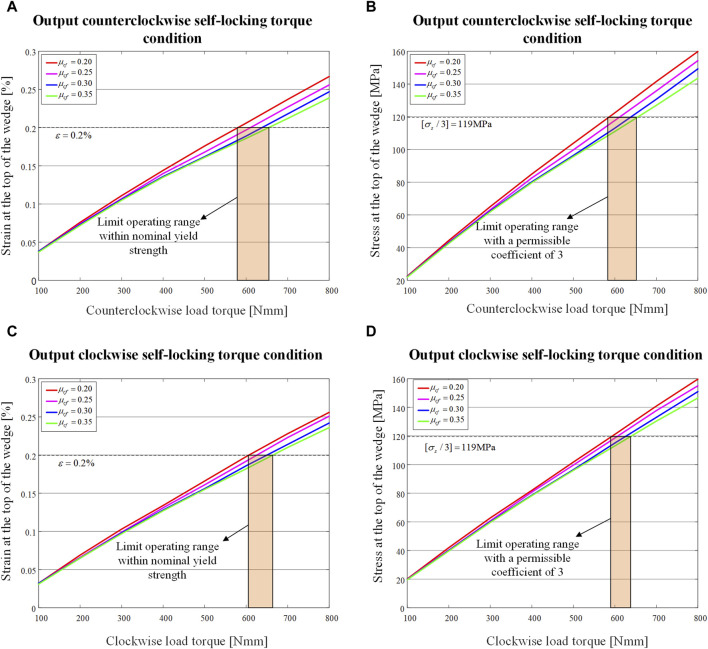
Maximum stress and maximum strain at the top of the wedge when load torque is applied to the output end for different values of $\mu_{cf}$ . **(A)** Maximum strain curve of the wedge when load torque is applied counterclockwise. **(B)** Maximum stress curve of the wedge when load torque is applied counterclockwise. **(C)** Maximum strain curve of the wedge when load torque is applied clockwise. **(D)** Maximum stress curve of the wedge when load torque is applied clockwise.

The maximum stress and strain at the top of the wedge for different values of $\mu_{cf}$ are plotted against the load are shown in Figure 11.

The overall trend is that as $T_{o}$ increases, the stress at the top of the wedge increases linearly, and as $\mu_{cf}$ increases, the maximum stress tends to decrease. Excessively large $\mu_{cf}$ often causes wear problems when the system works for a long time, so $\mu_{cf}$ = 0.25 is the preferred value. Bounded by a strain of 0.2% at the nominal yield stress, the bi-directional self-locking torque at the load side of the system is about 600 Nmm (greater than this value also allows self-locking, but carries the risk of irreversible deformation of the wedge, which needs to be verified experimentally). In the study by R Muraliraja et al. (Muraliraja et al., 2019), the ultimate yield stress of the 7,075 aluminum alloy is $\sigma_{s}$ = 357 MPa. When the strain is 0.197%, close to the nominal yield strength of 0.2%, the stress is 117.76 MPa, which is about 1/3 of the ultimate yield stress, meaning that the safety allowable factor is 3, as indicated in the stress-torque diagram in Figures 11B, D.

By drawing a boundary line $\left[\sigma_{s}/3\right]$ = 119 MPa, it can be found that the extreme working range of the self-locking system under this boundary condition is basically consistent with the working range under the ε = 0.2% condition (see in Figures 11A, C).

## 3 Experimental analysis of NBDC

**FIGURE 12:**
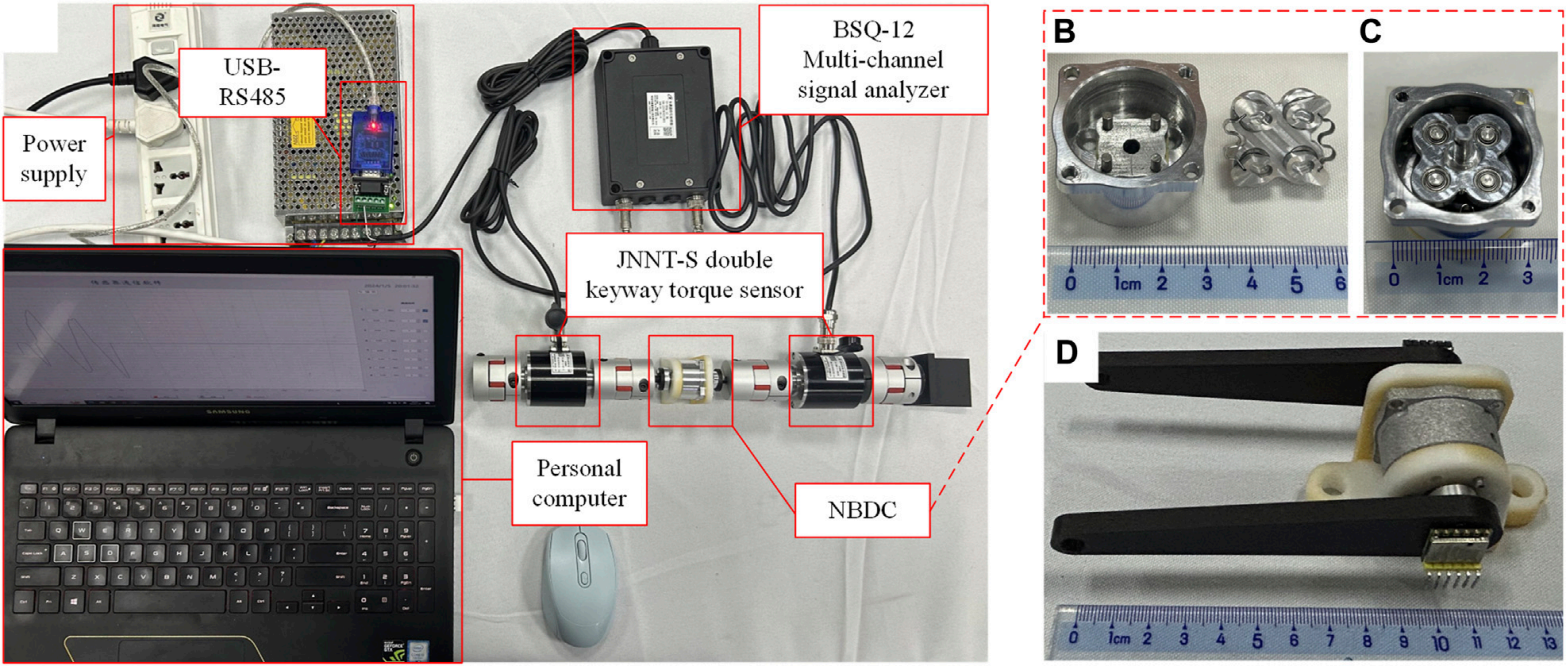
NBDC torque test experimental setup. **(A)** Experimental setup. **(B)** NBDC components. **(C)** Assembled NBDC. **(D)** NBDC unlocking test.

According to the previous analysis, a 3D model was established, and a prototype was assembled. The pin pedestal, wedge pedestal, wedges and fixed support are made of aluminum alloy (7,075 series), wavy springs were customized (stainless steel, 301 series, stiffness is around 0.30 N/mm), the four pins were selected as standard parts (stainless steel, 304 series, Φ 2.5 × 8), the four flange bearings are standard MF63ZZ 3 × 6 × 2.5 (bearing steel, 45#). The prototype is shown in Figures 12B, C, with a diameter of 33 mm and height of 15 mm, weighting 25.5 g.

The NBDC was positioned between two JNNT-S dual keyway torque transducers to test the unlocking torque from the input end and the maximum self-locking torque from the output end, which are shown in Figure 12A. The system configuration is as follows:

(1) The input end and output end were connected to the connecting flange through tightening screws, and then connected to the JNNT-S double keyway torque transducers through an elastic coupling (static torque transducer, movement range of 0°–180°, torque range of 0–20 Nm).
(2) JNNT-S double keyway torque transducer were connected to BSQ-12 multi-channel signal analyzer which connected to a computer via USB-RS485, outputting the synchronous torque of two transducers.
(3) Elastic coupling could eliminate the axis deviation of the overall transmission system, the transmission efficiency is 98%, which was negligible.
(4) By fixing the output end coupling, bidirectional torque could be transmitted to the input end, and the unlocking torque would be obtained through numerical analysis of the two sensors.
(5) By fixing the input end coupling, bidirectional torque could be exerted on the output end, and the maximum self-locking torque would be obtained through numerical analysis of the two sensors.

In order to test the bidirectional unlocking idle angle, a 10 cm long rocker was installed at the input end and the output end respectively to facilitate the experiment. The WT901164K angle sensors (sampling frequency is 2000 Hz) were placed at the center of the shafts to capture the angle changes of the two ports simultaneously, as shown in Figure 12D.

**FIGURE 13:**
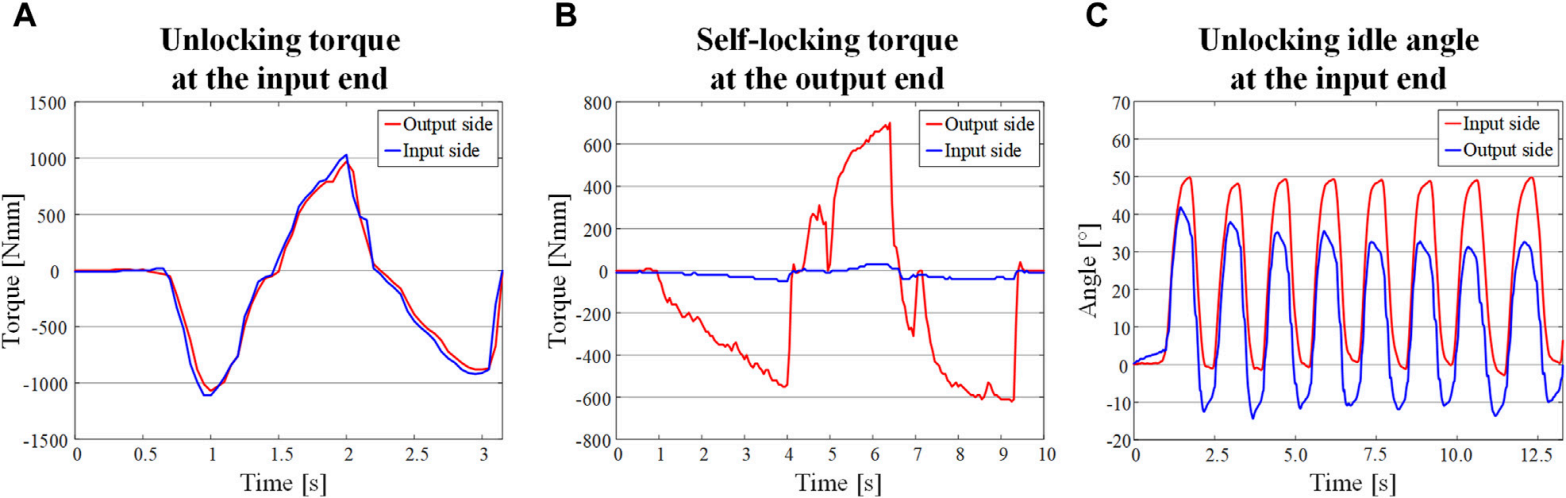
Unlocking and self-locking experiments of the NBDC. **(A)** Bidirectional unlocking torque change at the input end. **(B)** Self-locking torque change at the output end. **(C)** Unlocking idle angle at the input end.

By applying torque to the input and output ends respectively, the corresponding response was recorded by the torque sensor. As shown in Figure 13A, when the input end is unlocked, the output torque lags the input torque change and the difference is within 80 Nmm. This value is the unlock torque of the system, which is basically the same as the theoretical value in Figure 5D. From the results of transient dynamics, the self-locking torque of the system during normal operation is about 600 Nmm. When the load applied to the output is 600 Nmm, as shown Figure 13B, there is barely no torque response from the input end (less than 40 Nmm in both directions), which is consistent with the finite element simulation. The reason for the torque fluctuation at the input end is when the diagonal wedge self-locks, the other diagonal wedge rotates a slight angle, and during the rotation, the inner wall of the wedge groove transfers part of the force to the corresponding pin. The force is further transmitted to the torque sensor through the pin pedestal. This small torque will not cause back-driving phenomena at the input end. By rotating the rocker in both directions at the input end, it can be seen that the angular response of the output end is shifted by 14°, as shown in Figure 13C, which is consistent with design of the idle angle.

## 4 Applications with NBDC embedded in a prosthetic wrist

**FIGURE 14:**
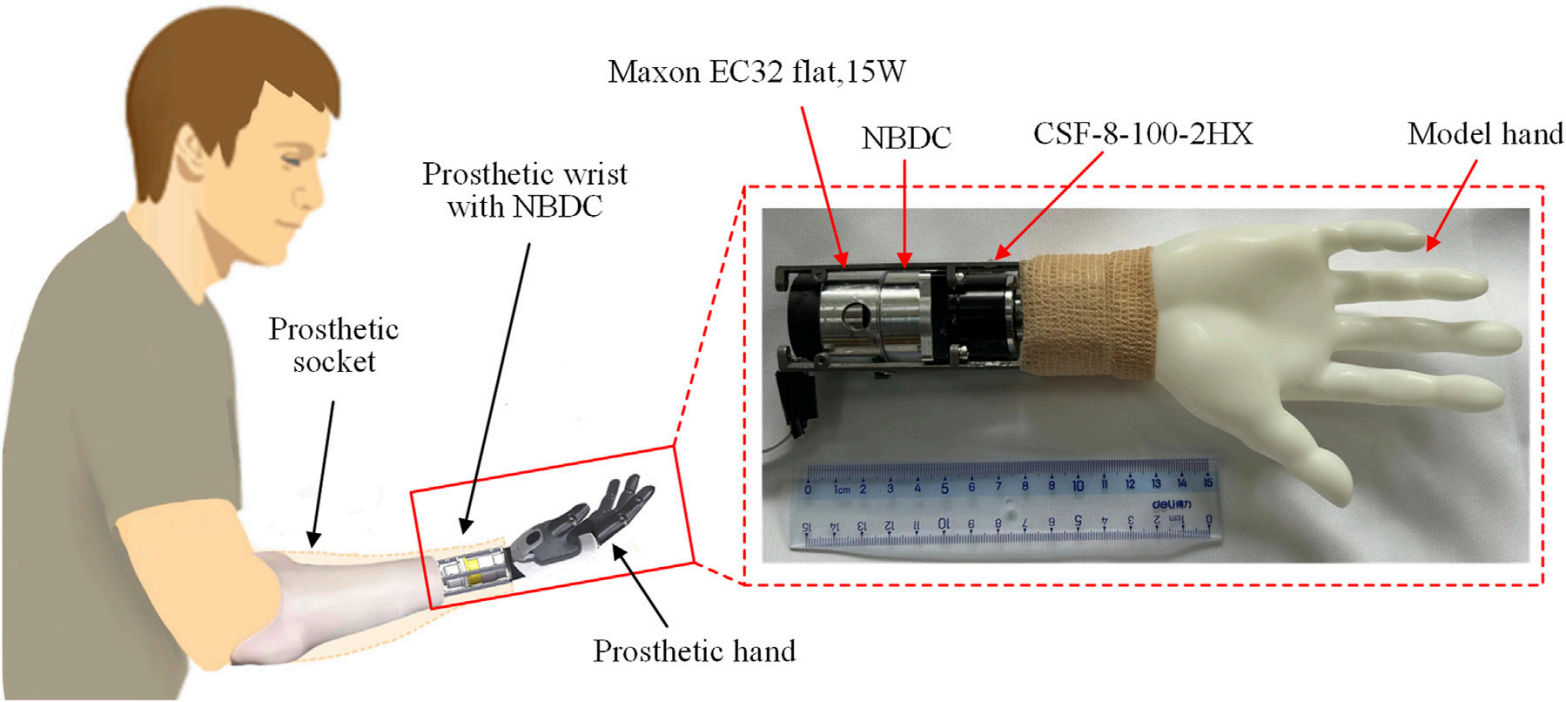
Schematic of the specific application of the NBDC mechanism to the prosthetic wrist.

To better demonstrate how the proposed component could be used in practical applications, a prosthetic wrist with rotation function was built, integrating the proposed NBDC. There are three reasons for the chosen of the application scenario: (1) The rotational degree of freedom of the wrist is mostly used in practice (Seo et al., 2017). (2) The rotation of the wrist is prone to stay unchanged once reach the target angle (Neumann, 2016). (3) The physiological rotational movement of the human wrist comes from the rotation of the radius around the ulna, and the actual rotational movement occurs near the elbow joint. If the amputation was executed near the hand, the amputee would still retain some rotational function of the forearm and does not need a rotationally functional prosthetic wrist. Thus, the prosthetic wrist module with rotational freedom is suitable for amputees with amputations close to the elbow, providing ample space for the implementation of the prosthesis. Figure 14 illustrates the application scenario where the proposed locking component was integrated to a prosthetic wrist. The prosthetic wrist was attached to the amputee through a prosthetic socket, and the constituent components of the prosthesis from the socket to the hand were the driving motor, the self-locking component and the harmonic reducer respectively. The integrating of the proposed locking component would allow the transmission of the rotational motion of the motor to the load transparently, while the time-varying torque generated at the load side would be prevented from transmitting back by this component, which ensures the stability of the prosthetic joint and reduces the power consumption. The component, with a diameter of 33 mm and a length of 15 mm, weighting 25.5 g, could theoretically be integrated in series to any forearm joint except the hand without compromising the total weight and dimension, and thus has the potential to be widely used in prosthetic rotary joints.

**FIGURE 15:**
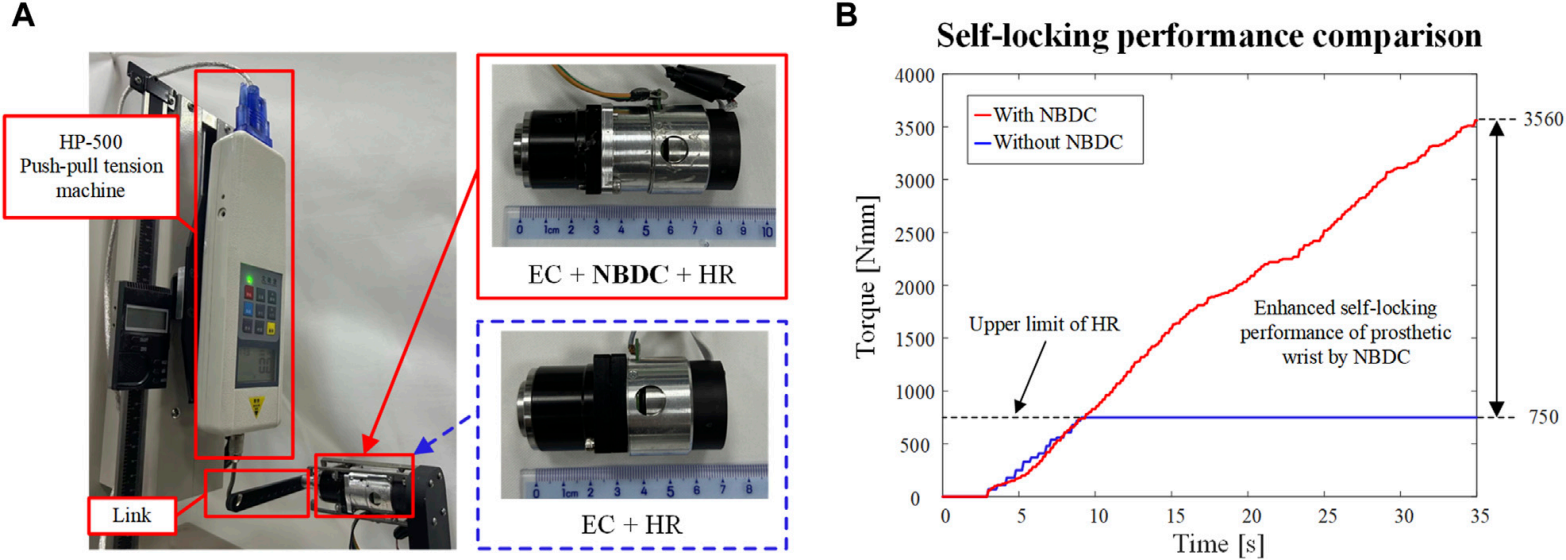
Experimental self-locking performance of NBDC in prosthetic wrist system. **(A)** The self-locking performance of the prosthetic wrist with and without the NBDC mechanism was tested separately. **(B)** Self-locking performance comparison.

Figure 15A illustrates the self-locking performance experiments of NBDC in a prosthetic wrist system, where the self-locking ability of the wrist joint was tested with and without NBDC, respectively, and the results are shown in Figure 15B. Since the harmonic reducer itself has certain self-locking capacity, the upper limit of the self-locking capacity of without the embedded NBDC component is 750 Nmm. By contrast, the self-locking capacity with the NBDC reaches 3560 Nmm in this experiment. It is worth pointing out that 3560 Nmm is not the upper limit of the system with the NBDC, but a safe experimental limit set to protect the harmonic reducer. From the calculation and analysis in Section 3, it can be seen that the theoretical upper limit could reach 600 × 100 = 60,000 Nmm under this condition. However, excessive loads would not occur in real application scenarios, and the improvement from 750 Nmm to 3560 Nmm could adequately demonstrate that the embedding of the NBDC mechanism could bring significant improvement in the self-locking performance of the rotary joint. The specific mechanical requirements and wearability requirements are summarized in Table 1.

**TABLE 1:** Mechanical requirements and wearability requirements for the NBDC mechanism.

Classification	Parameter	Unit	Value
Mechanical requirements	Required rotation angle	°	Not required
	Stiffness of the wavy spring	Nmm	0.27–0.33
	Unlocking torque of the input end	Nmm	65–80
	Maximum torque of the input end	Nmm	Not required
	Maximum torque of the output end	Nmm	Within 600
	$\mu_{cf}$	—	More than 0.25
Wearability requirements	Height	mm	15
	Diameter	mm	33
	Weighting	g	25.5

## 5 Discussion

This paper designed a new type of NBDC mechanism, theoretically analyzed the self-locking conditions of the model and the influence of different friction coefficients on the self-locking performance, revealed that the friction coefficient $\mu_{cf}$ should be at least 0.16, as shown in Eq. 26, which was verified in a transient kinematic finite element simulation shown in Figure 10. The current locking torque of the mechanism is still not ideal, which is mainly constrained by the materials of the two contact bodies. In this paper, 7,075 aluminum alloy is chosen due to the lightness, which is important for prosthetic wrists. If lightness is not the primary consideration, materials with higher yield strengths could be adopted to further increase the maximum locking torque. In the simulation for the performance of different $\mu_{cf}$ , as shown in Figure 11, a higher $\mu_{cf}$ is preferable to improve the locking torque, and the friction coefficient of the contact surfaces can be further improved by means of surface sandblasting, etc. However, the potential to improve the locking torque in this way is limited, so we chose $\mu_{cf}$ = 0.25 as a representative case. In terms of the surface contact form, line contact is currently adopted in this study, which limits the performance to a certain extent, where further improvement could be expected by expanding the contact area. For the number of wedges, it is possible to follow M. Controzzi’s research (Controzzi et al., 2017) by placing more wedges through dimensional optimization, further enhancing the self-locking performance. In terms of the idle angle, it only exists on the motor side, but not on the load side, which is to say, if the motor moves, it has to offset an idle angle before it starts to drive the load. The motion and force on the load side in any direction will not be transmitted to the motor side. In terms of the unlocking torque, the main constraint is the stiffness of the wavy spring. High stiffness will increase the unlocking torque and make it more difficult for the system to work properly. Low stiffness will potentially result in the failure of the spring or the inability to recover the self-locking state in the case of the maximum deformation. Therefore, the type of spring should be optimized in the future to reduce the unlocking torque of the system. During normal operation, driving from the motor requires a 14° idle rotation to trigger the unlocking state of the NBDC, which helps to reduce wear inside the mechanism, but would introduce a position error for the control system. Fortunately, this idle angle can be compensated by an external angle sensor.

## 6 Conclusion

In this paper, a novel NBDC is proposed, which can be applied in the forearm rotary motion joints of prosthetic wrists. It may save power to a certain extent under the premise of guaranteeing the safety and stability of manipulations. Detailed kinematic, static and transient dynamic analysis were carried out, and a prototype of the proposed design was assembled and tested to verify its self-locking and unlocking performance. Experimental results showed that the bidirectional self-locking torque of this NBDC for external load is about 600 Nmm, and the unlocking torque at the input end is about 80 Nmm. It is worth pointing out that connecting a reducer in series between the output end and the load can further expand the self-locking performance of the mechanism, thereby expanding its application scenarios.

## Data Availability

The original contributions presented in the study are included in the article/Supplementary material, further inquiries can be directed to the corresponding author.
